# Variable-fidelity EM analysis and simplex-anchored regression surrogates for efficient global optimization of microwave passive circuits

**DOI:** 10.1038/s41598-025-25969-3

**Published:** 2025-11-26

**Authors:** Anna Pietrenko-Dabrowska, Slawomir Koziel

**Affiliations:** 1 Faculty of Electronics, Telecommunications and Informatics, Gdansk University of Technology, 80-233 Gdansk, Poland; 2 Engineering Optimization & Modeling Center, Reykjavik University, 102 Reykjavik, Iceland

**Keywords:** Design automation, Global optimization, EM-based microwave design, Features, Simplex, Data-driven modeling, Engineering, Electrical and electronic engineering

## Abstract

Formal optimization is nowadays ubiquitous in microwave design. It is frequently conducted using electromagnetic (EM) simulations, which guarantee dependability. Yet, it is computationally expensive. Local tuning may involve hundreds of system analyses, whereas global EM-driven optimization typically generates unmanageable expenses. However, global search is often imperative, for example, in design of miniaturized components, large-scale operating frequency re-design, problems with multiple local optima (design of metasurfaces or frequency selective surfaces). This study suggests a procedure for low-cost globalized optimization of microwave circuits. Its keystones are simplex-based regression surrogates constructed to represent the circuit’s operating parameters. Geometrical simplicity of the surrogate and only a slightly nonlinear relation between the circuit dimensions and operating parameters, as well as conducting global search using low-resolution EM simulations, lead to a remarkable cost efficiency of the algorithm. Meanwhile, the assumed simplex updating rules guarantee convergence. The reliability is secured by a supplementary fine tuning executed using high-resolution EM models. As demonstrated, the presented framework exhibits perfect success rate with satisfactory designs found in each algorithm run out of multiple instances executed. The cost is just sixty high-resolution EM analyses, whereas design quality is competitive over the benchmark methods.

## Introduction

Design of modern microwave circuit design has become an increasingly intricate process due to performance and functionality demands, e.g., tunability, multi-band operation, harmonic suppression, etc^1–3^. Representative examples are miniaturized structures, where miniaturization techniques (e.g., transmission line meandering^4^, using compact resonant cells^5^ lead to complex structures involving numerous parameters^6^. Accurate evaluation of their characteristics and accounting for EM cross-coupling, losses, and/or auxiliary components (e.g., fixtures, housing, connectors) require electromagnetic (EM) analysis. For the same reasons, the design process, including the adjustment of circuit dimensions, must be EM-driven. While traditional approaches to geometry tuning (e.g., parametric studies) remain common, it is rigorous numerical optimization that enables concurrent handling of several variables and design goals. A common issue of EM-based optimization is excessive CPU cost. The overhead associated with local parameter adjustment may exceed hundreds of EM analyses. Other procedures, such as global search, generate much higher expenses, yet may be necessary in a many cases, e.g., optimization of metasurfaces^7^, array pattern synthesis^8^, multi-criterial design^9^, circuit re-design over wide frequency spectrum^10^, or compact circuit design.

Over the last decades, global optimization was primarily conducted using bio-inspired techniques^11,12^. Widely used algorithms include evolutionary algorithms^13^, particle swarm optimization (PSO)^14^, and differential evolution (DE)^15^. A plethora of new methods have been reported as well (e.g., grey wolf optimization, firefly algorithm, harmony search, and many others^16–18^. The ability to carry out global search is associated with information exchange between candidate solutions processed by the algorithm^19^, but also the presence of stochastic components, such as local changes (mutation)^20^ or randomized design relocation^21^. Nature-inspired techniques are easy to implement but exhibit poor computational efficiency: typically, thousands of objective function calls are necessary per algorithm run. Needless to say, EM-driven optimization using population-based methods is prohibitive, unless full-wave analysis is fast or available resources and licensing allow for parallelization. In practice, nature-inspired optimization is applied whenever analytical models are available (e.g., antenna array pattern synthesis^22^.

Surrogate modelling (kriging^23^, neural networks^24^, Gaussian process regression^25^ is a common approach to improving efficiency of global optimization^26,27^. Data-driven metamodels assume a role of fast predictors and are iteratively updated using the EM simulation results accumulated during the algorithm run (so-called infill points^28^. These methods fall into the machine learning category^29^. In some procedures, the surrogates are used for space pre-screening^30^. Delegating most operations to a low-cost surrogate may dramatically improve the efficacy. Notwithstanding, the building behavioral metamodels is impeded by dimensionality-related issues. For high-frequency circuits, additional difficulties arise due to response nonlinearity (typically, *S*-parameters versus frequency). Consequently, many machine learning schemes are illustrated using rather simplistic test cases (few parameters, narrow ranges thereof)^31^. The dimensionality-related difficulties may be alleviated using constrained modelling methods^32,33^. Therein, the surrogate is only rendered near the manifold encapsulating optimum designs^33^, which considerably reduces the number training points. Another option is utilization of response features^34^. Therein, restating the design problem using appropriate characteristic points regularizes the objective function, which translates to accelerated convergence^35^, or enhancing the surrogate’s predictive power^36^.

This work focuses on low-cost simulation-based global optimization of microwave circuits. A novel methodology is presented which employs regression models representing circuit’s operating parameters, constructed using the sets of affinely independent parameter vectors (simplexes), and relocated towards the optimum design in a convergence-guaranteeing manner. A weakly-nonlinear dependence of the operating conditions on system’s dimensions ensures superb dependability of the surrogates even when constructed using few training points. The efficacy is further improved by carrying it out using low-resolution EM simulations. The high-resolution model is only employed for final (gradient-based) parameter tuning to ensure evaluation reliability. The proposed approach is validated using several circuits and juxtaposed against a range of benchmark routines, including bio-inspired algorithms, randomized gradient search, and a surrogate-assisted algorithm with simplex-based predictors exclusively using high-resolution EM analysis. The results indicated that our algorithm performed competitively and consistently in terms of design quality and CPU efficiency. The typical optimization cost is just sixty EM simulations, less than the expenses incurred by direct local parameter adjustment. Global search capability, low running cost, and straightforward setup make the presented methodology an attractive alternative to existing EM-driven methods for microwave passive circuit design.

The original contributions of the work can be summarized as follows: (i) the development of a novel approach to global optimization of microwave circuits using leveraging regular relationships between the system’s operating parameters and design variables, (ii) conducting low-cost global search using simplex-based regressors constructed at the level of the operating parameters, (iii) accelerating the optimization process using variable-resolution EM models, (iv) demonstrating the exquisite performance of the presented framework and its superiority over several state-of-the-art benchmark methodologies.

## Globalized microwave optimization using Simplex-Based predictors and variable-resolution EM analysis

The suggested methodology for global parameter adjustment of microwave passives is explained in this section. First, we state the EM-driven design task and discuss multi-resolution EM models (Sect. 2.1 and Sect. 2.2). The formulation of simplex-based regression models is elucidated in Sect. 2.3, whereas Sect. 2.4 and 2.5 elaborate on the key search stages of the framework, respectively. Section 2.6 puts together the complete algorithm.

### EM-driven microwave optimization

**Fig. 1:**
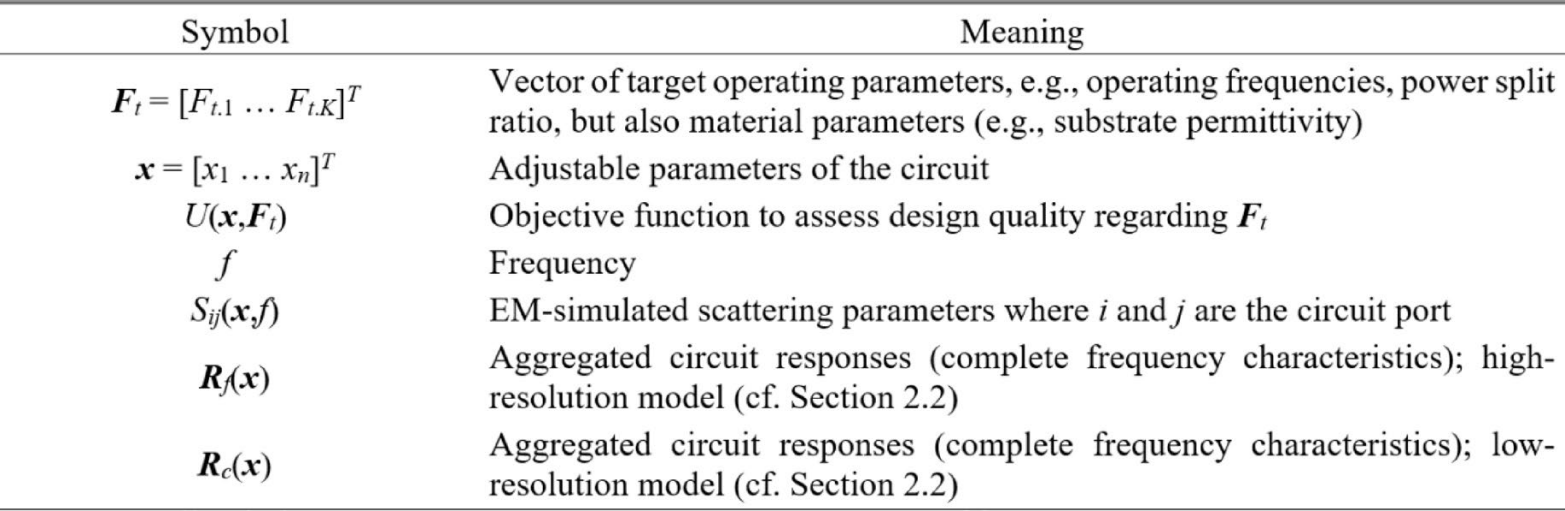
Basic notation used in this study.

**Fig. 2:**
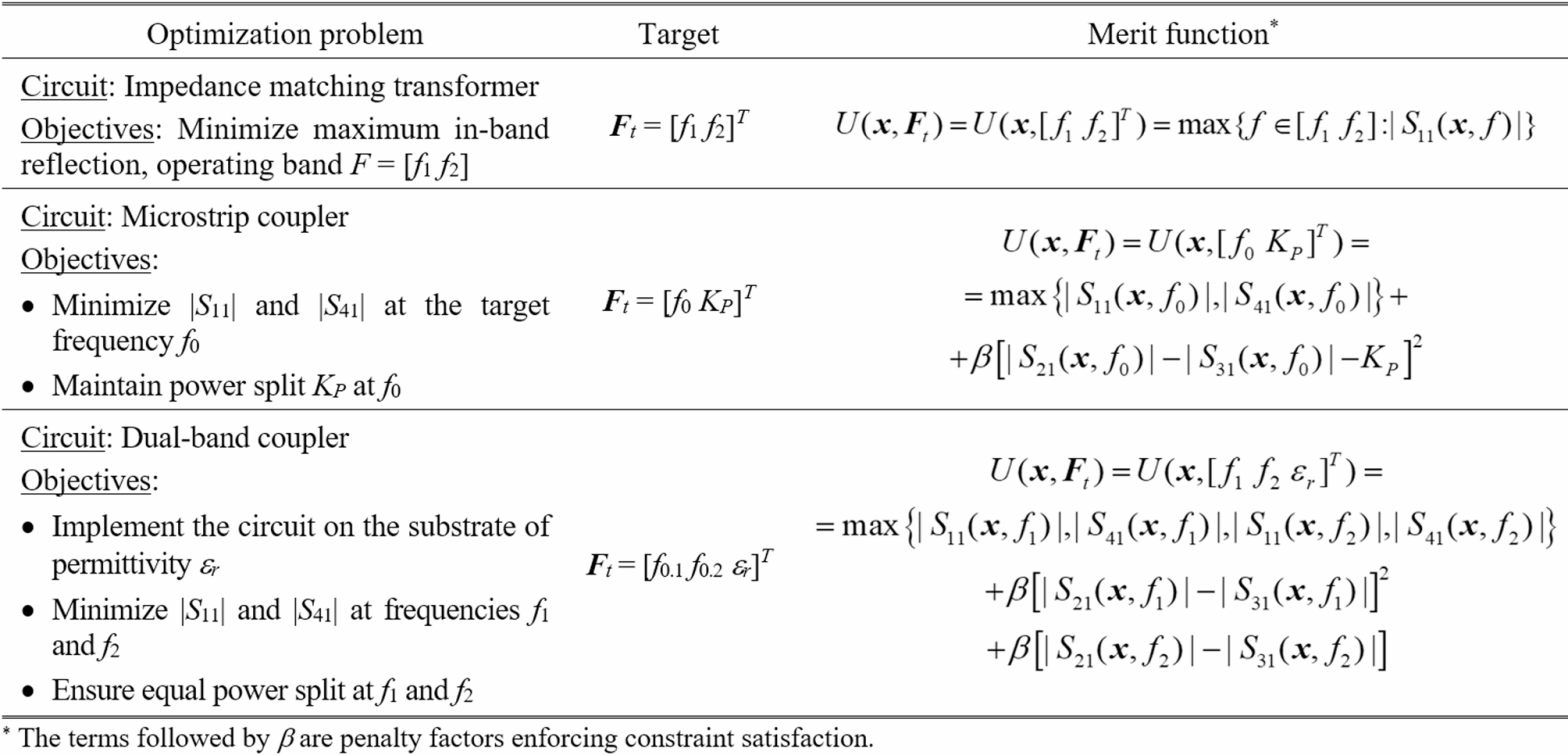
Microwave optimization problems: selected examples.

Figure 1 explains the notation used throughout the paper. The central concept is a merit function, which is determined so that higher-quality designs are assigned lower values of *U*(***x***,***F***_*t*_). The function *U* is scalar. In case of multiple design goals, a primary objective is selected and treated directly, whereas the remaining ones are stated as constraints and controlled using penalty functions^37^. This has been shown in Fig. 2, which provides a number of examples of microwave optimization tasks along with the formulations of *U*(***x***,***F***_*t*_). Other techniques are objective aggregation^38^, or multi-criterial optimization^39,40^, which is out of the scope of this work.

Following the terminology contained in Fig. 1, the EM-driven parameter adjustment problem is posed as


1


 Here, the optimum parameter vector to be identified has been marked as x*.

### Variable-resolution EM analysis

**Fig. 3:**
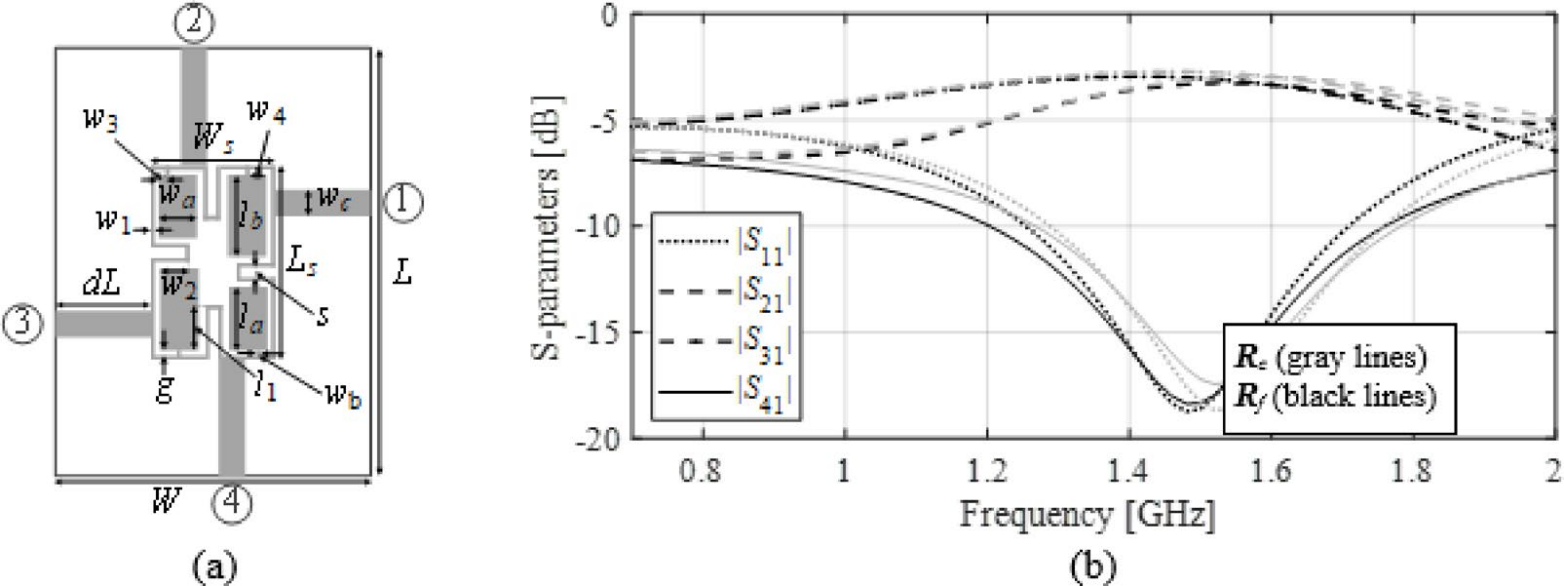
Multi-resolution models: (**a**) a miniaturized coupler, (**b**) *S*-parameters. Here, ***R***_*f*_ and ***R***_*c*_ simulate in 250 and 90 s, respectively.

Multi-fidelity modelling concept was used for quite a long time. Traditionally, the low-fidelity (or coarse) representation is either analytical description or equivalent network^41^, whereas high-fidelity (or fine) model is understood as EM analysis. In the realm of design automation, a more convenient approach is variable-resolution modelling, where the fidelity of system evaluation is controlled by altering the structure discretization density within EM simulation (see^42^ for other options). It allows for using the same underlying representation and exploring the compromise between computational costs and the precision of rendering circuit responses, cf. Figure 3. In practice, the accuracy loss due to relaxing model resolution can be accommodated through appropriate correction^43^. If the low-resolution model is to account for all essential features of the circuit characteristics, the resolution cannot be reduced excessively. For typical microwave passive components, the acceleration factor varies from less than three to about six. Although most practical schemes use two levels of resolution^44^, recently, model management schemes involving a continuous spectrum of model fidelities have been reported^45^.

In this paper, we explore two resolution levels: the low- and high-fidelity models ***R***_*c*_(***x***), and ***R***_*f*_(***x***). ***R***_*c*_ will be employed for three purposes: (i) initial parameter space sampling, (ii) construction of the regression model, and (iii) conducting the global search stage. These steps involve a considerable number of circuit evaluations. Consequently, carrying them out at the level of ***R***_*c*_(***x***) will lead to noticeable computational savings. The model ***R***_*f*_(***x***) will be used in the last step, which is final (gradient-based) parameter tuning. This stage is relatively inexpensive because the starting point generated through global search is (hopefully) close to optimum. Nevertheless, using high-resolution model is imperative to ensure circuit evaluation accuracy.

It should be emphasized that the low-fidelity model is set up to properly represent the relevant features of the system outputs. In this work, the model is selected manually, using a grid convergence study. In particular, the EM simulation is performed using different discretization densities of the circuit of interest, controlled by a solver-specific parameter (e.g., lines-per-wavelength in the case of CST Microwave Studio). Visual inspection is used to decide the specific model setup. One of the goals of the future work will be to automate the process, using, e.g., correlation analysis for the model simulated at a set of different designs.

### Simplex-anchored regression models

Appropriate tuning of circuit dimensions is imperative to achieve the best performance. Reliability requires that the process employs EM analysis. In a growing number of cases, global optimization is imperative (e.g., unavailability of a good initial point, problem multimodality), which is immensely challenging due to typically large parameter spaces to be explored. Surrogate-assisted techniques^46–48^ can be used to alleviate some of the associated difficulties. However, constructing accurate data-driven models of highly nonlinear microwave circuit responses is a serious bottleneck of these procedures.

#### Global optimization: operating parameters perspective

**Fig. 4:**
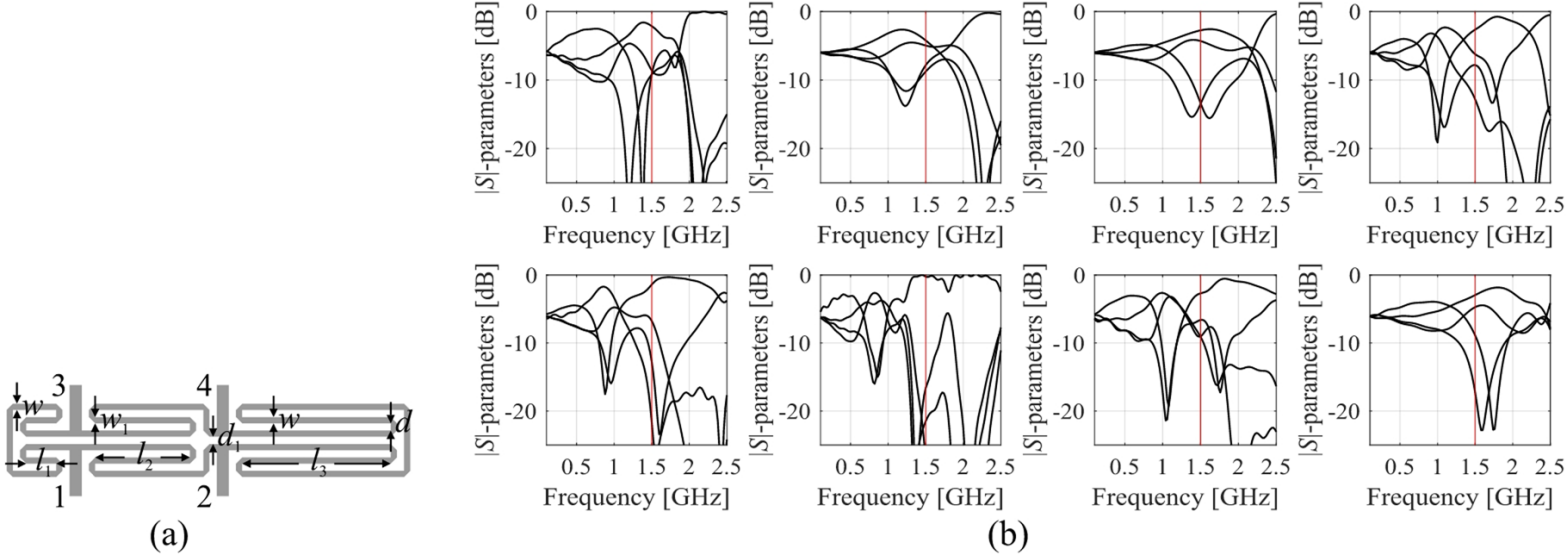
Compact coupler: (**a**) structure architecture, (**b**) *S*-parameters at randomly generated designs. An exemplary target frequency of 1.5 GHz is represented by the vertical line.

Here, we discuss the challenges pertinent to modelling and optimization of microwave circuits and advocate handling the task using operating parameters instead of complete frequency characteristics. As an illustration, consider a coupler illustrated in Fig. 4, and its EM-evaluated *S*-parameters several random designs.

Assuming a target frequency of *f*_0_ = 1.5 GHz, local optimization of the circuit (here, to minimize matching/isolation at *f*_0_ and achieving a target power division ratio *K*_*P*_) would be unsuccessful when started from most of the designs in (Fig. 4b). Similarly, building a reliable data-driven model of the *S*-parameters appears to be extremely difficult.

**Fig. 5:**
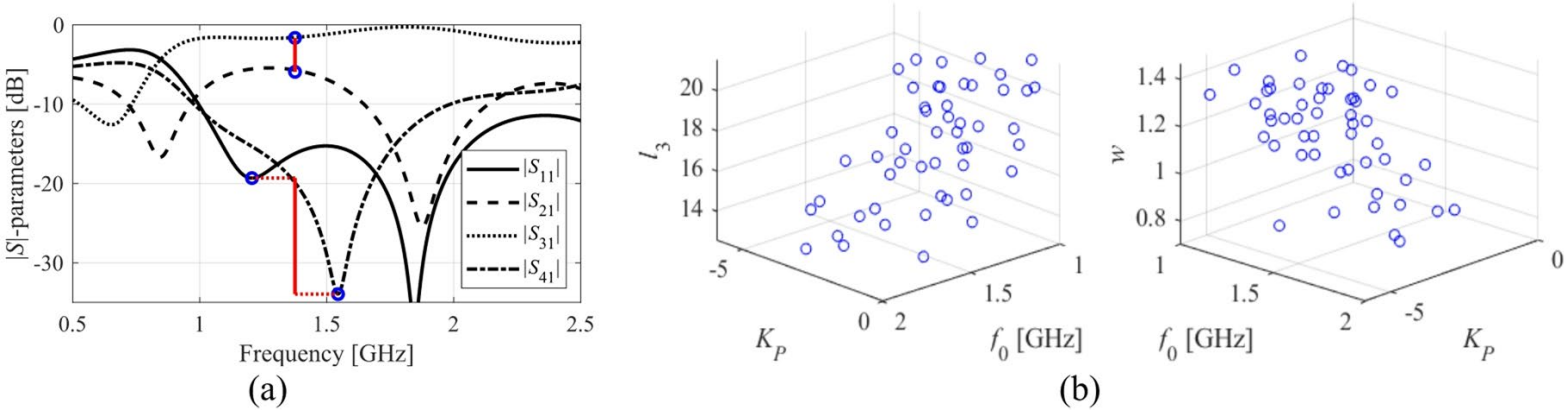
Operating parameters for the coupler of Fig. 4: (**a**) operating parameters: power division ratio *K*_*P*_ is computed at the operating frequency *f*_0_ taken as the average frequency corresponding to the |*S*_11_| and |*S*_41_| minima; (**b**) dependence of the operating parameters on selected circuit dimensions.

The situation is entirely different when considering the operating conditions, see Fig. 5. The relation between the system’s geometry and the two operating parameters (center frequency, power division ratio) is quite regular. The plots in Fig. 5b are obtained using parameter vectors, which are a subset of samples for which it was possible to extract the operating parameters. Even though the said designs are not optimized in any way, regular patterns can be observed, which are considerably simpler than the original frequency characteristics. As indicated in the literature, particularly the works concerning feature-based modelling and optimization^49,50^, this type of dependency is rather universal.

#### Simplex-based regression models. preliminary considerations

The variable-resolution global optimization methodology introduced in this work explores the relationships discussed in Sect. 2.3.1 (cf. Figure 5). As these dependencies are regular (essentially monotonic), their representation in the form of data-driven surrogate models can be made simple as well. A minimum condition would be to account for the number of circuit parameters *n*. Consequently, one needs *n* + 1 affinely independent points ***x***^(*j*)^ serving as the training dataset. The minimal geometrical object that can be spanned using such a set is a simplex. At the same time, using conventional data-driven modeling methods such as kriging, neural networks, etc., is unnecessary.

**Fig. 6:**
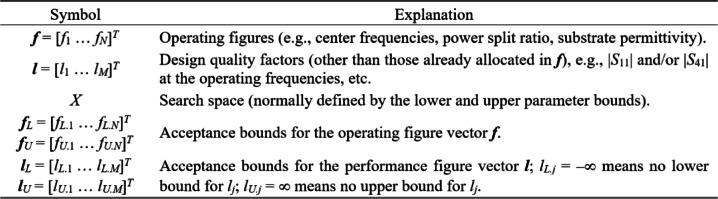
Notation used in the context of simplex-based regression models.

**Fig. 7:**
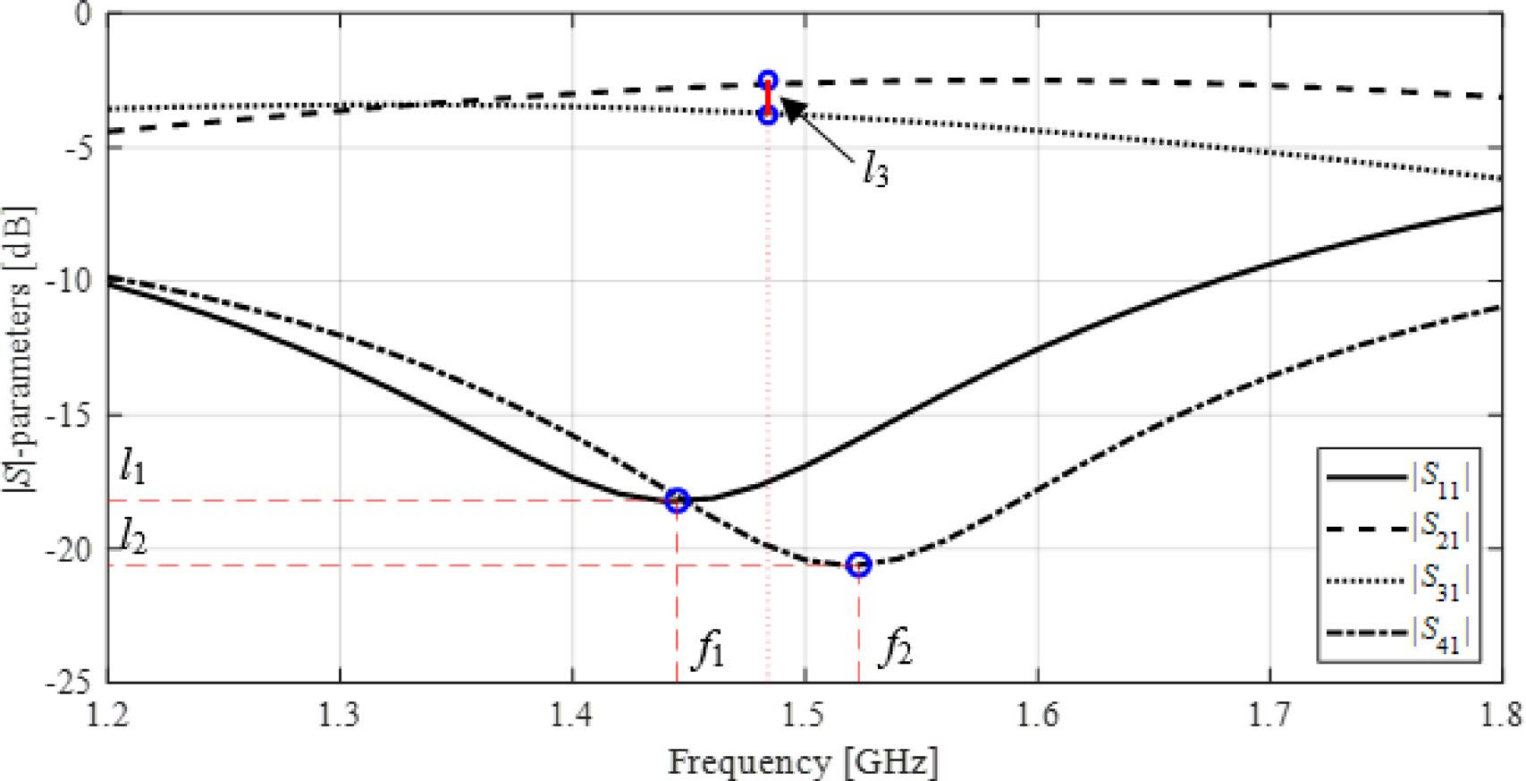
Operating and performance vectors ***f*** and ***l*** for a coupler: ***f*** = [*f*_1_ *f*_2_]^*T*^ (frequencies associated with the minima of |*S*_11_| and |*S*_41_|); ***l*** = [*l*_1_ *l*_2_ *l*_3_]^*T*^, where *l*_1_ and *l*_2_ are |*S*_11_| and |*S*_41_| at *f*_1_ and *f*_2_, respectively, whereas *l*_3_ is the power division ratio at (*f*_1_ + *f*_2_)/2.

In the following, we discuss a definition of a simplex-based regression surrogate and how it will be used in the context of circuit optimization. Figure 6 gathers the terminology to be used in our subsequent considerations. Figure 7 illustrates the operating and performance vectors ***f*** and ***l*** for a microwave coupler.

Let us consider *n* + 1 affinely independent points ***x***^(*j*)^ = [*x*_1_^(*j*)^ … *x*_*n*_^(*j*)^]^*T*^ in the space *X*, *j* = 0, …, *n*. The associated operating figure vectors and performance figure vectors are ***f***^(*j*)^ = ***f***(***x***^(*j*)^) = [*f*_1_^(*j*)^ … *f*_*N*_^(*j*)^]^*T*^ and ***l***^(*j*)^ = ***l***(***x***^(*j*)^) = [*l*_1_^(*j*)^ … *l*_*M*_^(*j*)^]^*T*^, respectively. All points ***x***^(*j*)^ are such that ***f***_*L*_ ≤ ***f***^(*j*)^ ≤ ***f***_*U*_, and ***l***_*L*_ ≤ ***l***^(*j*)^ ≤ ***l***_*U*_, for *j* = 0, …, *n*. In practice, the set {***x***^(*j*)^} is obtained by sequential (random) sampling and analyzing their corresponding ***f*** and ***l*** vectors. Only the samples satisfying the mentioned conditions (including the affine independence condition) are included into the set, others are rejected. The sampling process is stopped as soon as *n* + 1 vectors are found.

#### Simplex-based regressors

Let ***x*** ∈ *X*. Consider an expansion of ***x*** w.r.t. {***x***^(*j*)^}_*j*=0,…,*n*_ discussed in Sect. 2.3.2.


2


Because {***x***^(*j*)^} is affinely independent, ***x***^(*j*)^ – ***x***^(0)^ are linearly independent. This implies uniqueness of (2), so that ***a*** = [*a*_1_ … *a*_*n*_]^*T*^ is found as


3


 in which


4


 is an invertible square matrix of size *n* × *n*.

Our goal is to construct two types of data-driven regression surrogates, one predicting the operating parameters, ***F***(***x***) : *X* → *F*, and another one predicting the performance parameters, ***L***(***x***) : *X* → *R*^*M*^. ***F***(***x***) and ***L***(***x***) are built based on the set {***x***^(*j*)^} and the associated vectors ***f***(***x***^(*j*)^) and ***l***(***x***^(*j*)^). The formal definitions are as follows.


5



6


The coefficient vector ***a*** is obtained from (3), whereas the matrices ***X***_*f*_ and ***X***_*l*_ are defined as


7



8


The models (5) and (6) are interpolative with respect to the simplex vertices, that is, ***F***(***x***^(*j*)^) = ***f***^(*j*)^ and ***L***(***x***^(*j*)^) = ***l***^(*j*)^ for *j* = 0, …, *n*.

### Global optimization stage using simplex-anchored regressors

The models ***F***(***x***) and ***L***(***x***) aid the global search. Here, we leverage a regular relationship between circuit dimensions and the circuit’s operating parameters (cf. Figure 5). The latter greatly improves the predictive power of ***F***(***x***) and ***L***(***x***) over relatively large regions (as compared to conventional surrogates constructed to represent complete frequency characteristics), thereby allowing its efficient exploration.

#### Design quality

Quantification of circuit performance is fundamental for any optimization process. The original task (1) employs the objective function *U*, minimization of which is our ultimate goal. However, during global search, we aim to align the operating parameters ***f***(***x***) with the target ***f***_*t*_. Formally, ***f***_*t*_ and the target vector ***F***_*t*_ are not the same, but ***f***_*t*_ is derived from ***F***_*t*_ (although in some cases the vectors are the same). For clarification, let us go back to the coupler of Fig. 4. If the system is designed to work at a center frequency *f*_0_ and to produce a power division ratio *K*_*P*_, we have ***F***_*t*_ *=* [*f*_0_ *K*_*P*_]^*T*^. However, ***f*** = [*f*_1_ *f*_2_]^*T*^ (cf. Fig. 7). To ensure consistency, we need to set ***f***_*t*_ = [*f*_0_ *f*_0_]^*T*^.

Using the above concepts, the design quality will be assessed using the objective function *U*_*F*_ given by (here, *β*_*F*_ is a penalty factor).


9


Note that *U*_*F*_ depends on both ***f***(***x***) and ***l***(***x***). *U*_*L*_ is an auxiliary object introduced to quantify the performance parameters ***l***(***x***) and can be established as the function *U* of (1). For example, suppose that ***l***(***x***) = [*l*_1_ *l*_2_ *l*_3_]^*T*^ for a coupler structure considered before, cf. Fig. 7, where *l*_1_ and *l*_2_ are |*S*_11_| and |*S*_41_| at their respective minima, and *l*_3_ stands for *K*_*P*_ at the center frequency. Assuming that we aim to improve |*S*_11_| and |*S*_41_| while maintaining the required *K*_*P*_,. It is important to emphasize that *U*_*L*_ does not need to exactly match *U*(***x***). In general, the global search stage aims at reducing || ***f***(***x***) – ***f***_*t*_||, which is facilitated using the penalty factor in (9).

#### Global optimization: updating simplex

The regression models ***F***(***x***) and ***L***(***x***) predict ***f***(***x***) and ***l***(***x***) during the global search stage, which aims at minimizing the function *U*_*F*_. Accordingly, the candidate design ***x***_*tmp*_ is generated by solving a constrained task.


10


The following conditions are imposed on (10).


11



12


where *α* > 0 is a small number (e.g., *α* = 0.2). The condition (11) ensures that solution to (11) is restricted to the hyper-plane spanned by the vertices {***x***^(*j*)^}_*j* = 0,…,*n*_, whereas (12) confines the search region to the small neighborhood of {***x***^(*j*)^}. An additional condition is imposed upon the optimization process (10), which is that the sample set consisting of all simplex vertices but the worse one (cf. (14) below) and ***x***_*tmp*_ is affinely independent.

For convenience, ***x***^(*j*)^ are arranged so that ||***f***^(0)^ – ***f***_*t*_|| ≤ ||***f***^(1)^ – ***f***_*t*_|| ≤ … ≤ ||***f***^(*n*)^ – ***f***_*t*_||. Problem (10) is solved starting from ***x***^(0)^ (the best available design). The simplex is updated depending on the quality of ***x***_*tmp*_ produced by (10). We have:

- Design acceptance: the necessary condition for acceptance of the vector ***x***_*tmp*_ is that it improves over at least one ***x***^(*j*)^, i.e., the following inequality holds:

13


Vertex replacement: assuming that ***x***_*tmp*_ has been accepted, it replaces ***x***^(*jworst*)^, where


14


Simplex reduction: assuming that ***x***_*tmp*_ has been rejected, the simplex is diminished towards ***x***^(0)^ as follows:


15


where *γ* = 0.5 (a control parameter). Note that the reduction process leaves the best vertex ***x***^(0)^ intact.

If ***f***(***x***) is continuously differentiable, reducing the simplex size towards ***x***^(0)^ as in (15) generally improves the remaining vertices, i.e., leads to diminishing the norms ||***f***^(*j*)^ – ***f***_*t*_||. Furthermore, it can be shown that the vector ***x***_*tmp*_ generated by solving (10) will satisfy (13), assuming that the simplex is sufficiently small. The details are omitted for brevity.

Upon updating the simplex, ***F***(***x***) and ***L***(***x***) are refined accordingly, and used in the subsequent iteration. Global search is stopped if either of the three conditions listed below has been fulfilled (here, D = max {*j* $\in$ {1, 2,...n}: || x^(*j*)^ – x^(0)^||} is the simplex size):

- A design has been found such that

16


where *F*_max_ is a control parameter;

- The budget of *N*_*global*_ EM analyzes has been reached, *N*_*global*_ being a control parameter;
- *D* < *D*_min_ (a user-defined threshold).

Note that the termination does not guarantee the identification of a design that satisfies (16), as such a design may not exist for a given problem. Because global search is complemented by local parameter adjustment (cf. Section 2.5), the threshold *F*_max_ should be set so that the optimum is reachable from the design ***x***_*tmp*_ satisfying (16). In practice, *F*_max_ is a fraction (e.g., 50%) of the circuit’s bandwidth.

**Fig. 8:**
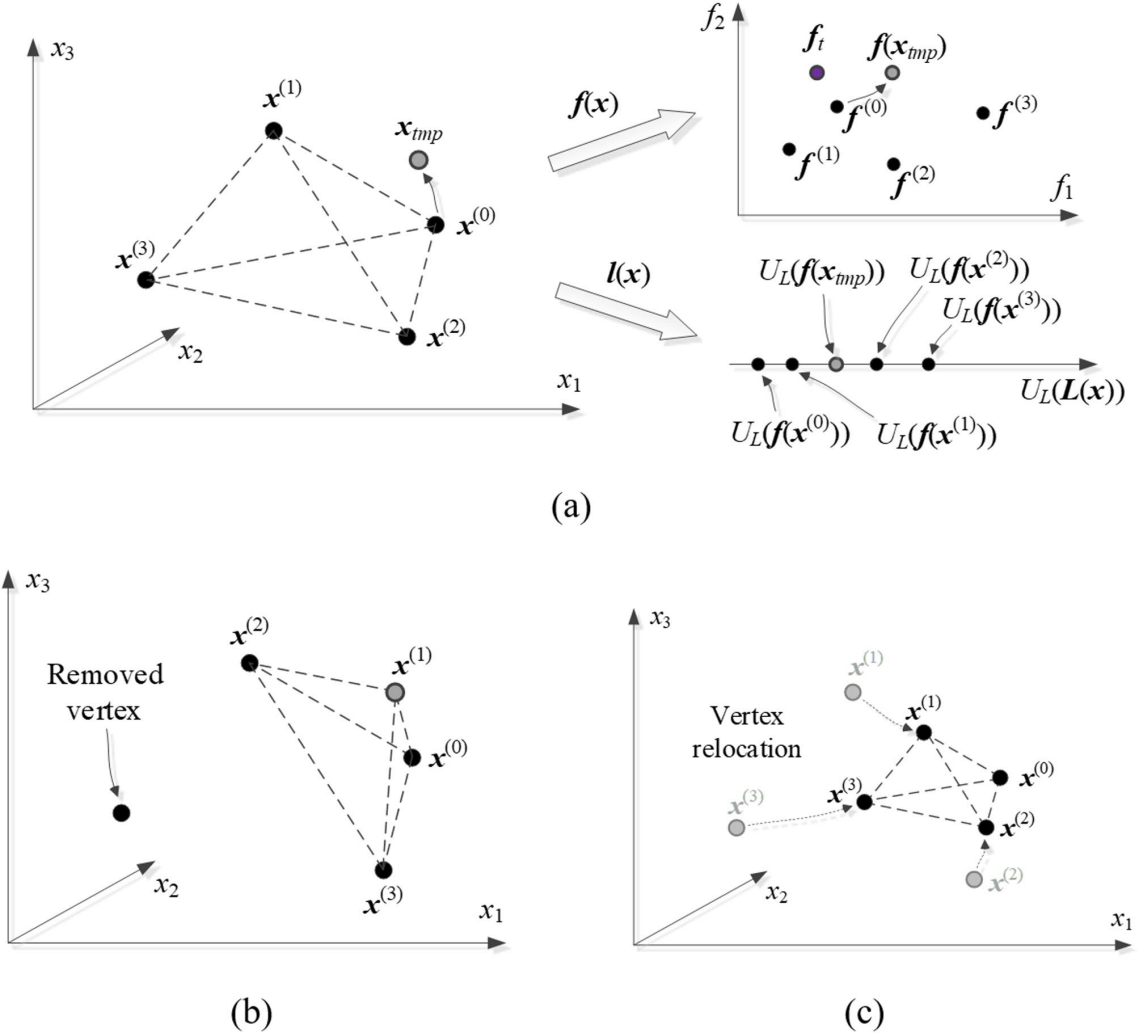
Global search: (**a**) current simplex. The predictions obtained using ***F***(***x***) and ***L***(***x***) are juxtaposed against ***f***(***x***) and ***l***(***x***) at ***x***_*tmp*_ obtained by (10). Here, ***x***_*tmp*_ will be accepted as it improves *U*_*L*_ and ||***f***(***x***) – ***f***_*t*_||; (**b**) corresponding simplex update (illustrated assuming that ***x***_*tmp*_ is better than ***x***^(1)^ but inferior to ***x***^(0)^); (**c**) simplex reduction if ***x***_*tmp*_ was rejected.

Figure 8 shows a graphical explanation of the simplex updating rules and a procedure for generating the candidate design ***x***_*tmp*_ using ***F***(***x***) and ***L***(***x***).

A comment regarding the extraction of the operating parameters from the circuit frequency characteristics should be added. A safety mechanism is incorporated into the procedure as follows: in case the operating parameter extraction fails (e.g., due to poor-quality of the design and/or heavily distorted responses), a high-value of the objective function is assigned (or large value of the norm ||***f***(***x***_*tmp*_) – ***f***_*t*_|| at the global search stage discussed in this section), which leads to the rejection of the said design.

### Local tuning

Global search is intended to identify a design close to the optimum in terms of the alignment of the operating parameters. Because this process does not directly minimize original merit function *U*, a final tuning is required. Here, it is implemented with the trust-region (TR) algorithm. The initial approximation is ***x***^(0)^ the outcome of the global search elaborated in Sect. 2.4.

The TR algorithm produces approximations ***x***^(*i*)^, *i =* 0, 1, …, to the vector x^*^ (cf. (1)). We have.


17


The analytical formulation of *U*_*L*_ is the same as *U* (see Fig. 2 for specific examples), but *U*_*L*_ is computed using a linear model ***L***^(*i*)^(***x***,*f*). If the characteristic of interest is *S*_*kl*_(***x***,*f*) (cf. Fig. 1), we have


18


 where ∇_*S*_ is found using finite differentiation (FD)^51^. The range parameter *d*^(*i*)^ is updated using the conventional TR rules, i.e., increased if the gain ratio


19


 is near unity, and reduced if *r* is close to zero or negative^51^. ***x***^(*i*+1)^ is accepted if *r* > 0, which is equivalent to *U*(***x***^(*i*+1)^,***F***_*t*_) < *U*_*P*_(***x***^(*i*)^,***F***_*t*_); otherwise, the next iteration starts again from ***x***^(*i*)^ with reduced *d*^(*i*)^.

Estimating gradients is the most expensive stage of the TR algorithm as it requires *n* EM analyses (*n* is the number of the circuit’s parameters). For the sake of acceleration, FD is replaced by a Broyden formula^52^ when close to convergence, i.e., ||***x***^(*i*+1)^ – ***x***^(*i*)^|| < *M*_*c*_*ε*, where *ε* is the required search process resolution (*M*_*c*_ = 10 is a control parameter). For alternative acceleration methods see, e.g^53^. , . Final tuning is ceased if ||***x***^(*i*+1)^ – ***x***^(*i*)^|| < *ε* or *d*^(*i*)^ < *ε* (here, *ε* = 10^–3^).

### Complete framework

**Fig. 9:**
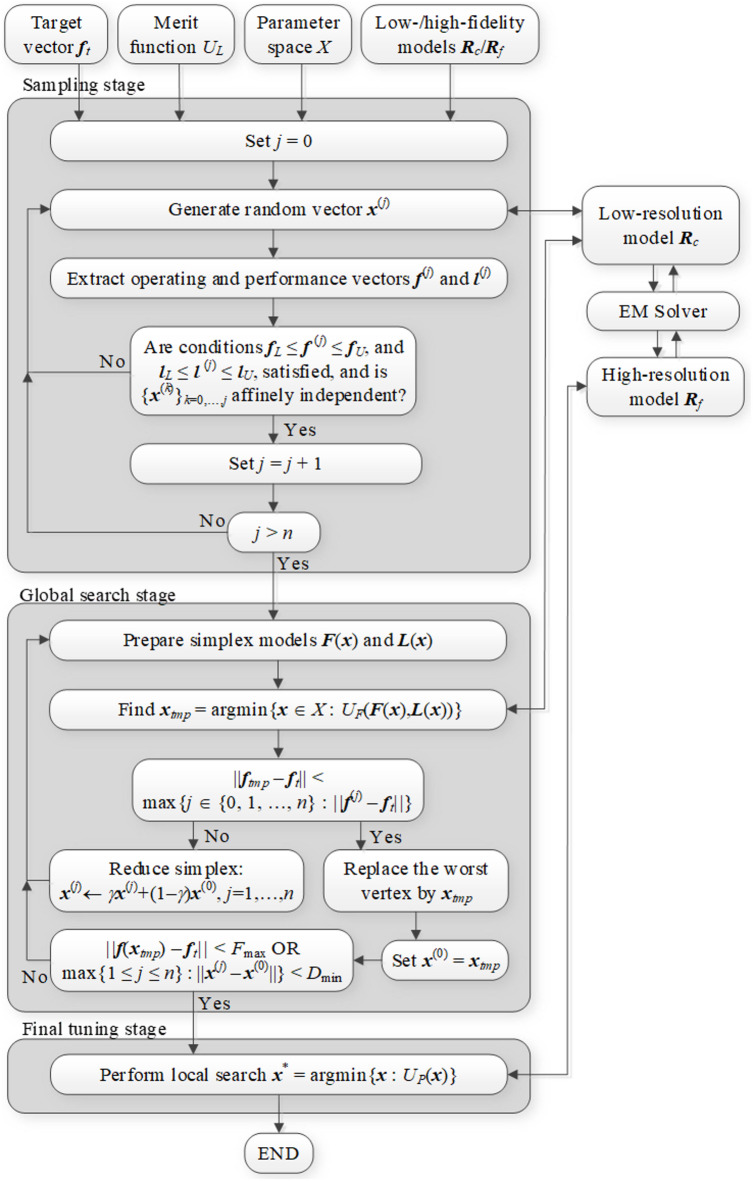
Proposed optimization algorithm: flow diagram.

**Fig. 10:**
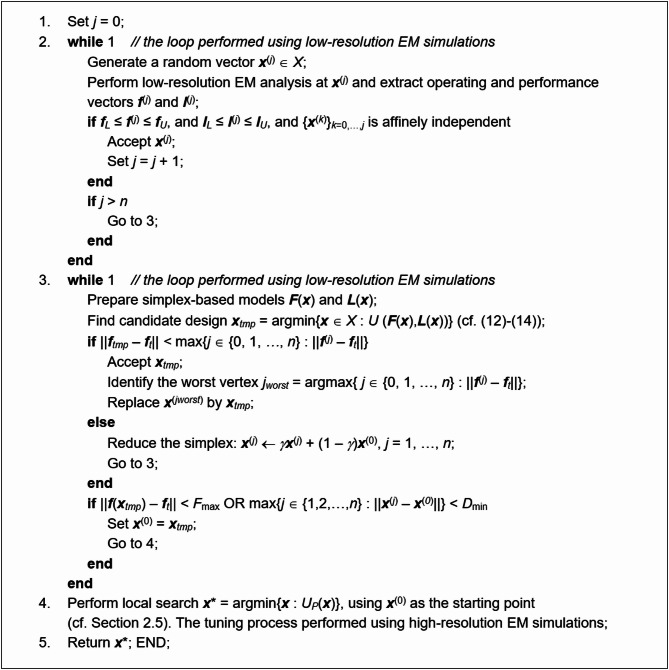
Proposed optimization algorithm: pseudocode.

Our optimization algorithm employs the algorithmic components discussed in Sect. 2.1 through 2.5. The input variables of the search process have been summarized in Table 1, whereas Table 2 gathers its control parameters. Figure 9 provides the flow diagram. It describes in detail all steps except final tuning, which was elaborated in Sect. 2.5. For additional clarification, Fig. 10 provides the pseudocode of the algorithm.

One of the important merits of the presented approach is straightforward setup. Among the control parameters listed in Table 2, only one, *F*_max_, is problem-dependent (see Sect. 2.4.2 for recommendations). Other parameters are mainly affecting the resolution of the search process. There is no need to adjust them for any given problem. To demonstrate this, the same algorithm setup is employed for all case studies of Sect. 3.

## Algorithm verification and benchmarking

The algorithm of Sect. 2 is showcased using several microwave circuits. Furthermore, it is compared to a range of state-of-the-art methods such as bio-inspired optimizers (here, PSO^54^ and differential evolution, DE^20^, randomized local search, and a simplex-based procedure operating using high-resolution EM simulations. The algorithm features to be verified include global search capability, design quality, as well as computational efficiency. This part of the manuscript is organized as follows. Section 3.1 and 3.2 outline the test cases and the benchmark procedures. Section 3.3 summarizes and discusses the results, as well as juxtaposes the proposed approach and the benchmark.

**Table 1:** Proposed optimization algorithm: input variables.

Parameter	Symbol	Comment
Search space	*X*	Interval delimited by the geometry parameters bounds
Objective function	*U*	User-defined function determining the design quality w.r.t. the target vector ***F***_*t*_
Computational models	***R***_*c*_, ***R***_*f*_	Low- and high-resolution EM models, respectively (cf. Section 2.2)
Operating/performance vectors	***f***, ***l***	Problem-dependent quantities for estimating operating parameters (***f***) and performance figures (***l***) as described in Sect. 2.3.2
Target vector	***f***_*t*_	User-defined quantities determining performance specifications, cf. Section 2.4.1
Merit function	*U*_*L*_	User-defined function for evaluating design quality (global search)

**Table 2:** Proposed optimization algorithm: control parameters.

Symbol	Explanation	Value
*F*_max_	User-defined threshold for terminating global search stage (cf. (16))	Problem-dependent (cf. Sect. 2.4.2)
*α*	Search region extension parameter (cf. (12))	0.2
*γ*	Simplex reduction ratio (cf. (15))	0.5
*D*_min_	Termination threshold for global search stage (minimum simplex size)	1% of the search space size
*N*_*global*_	Computational budget for the global search stage	Depending on available computational resources, typically set to 100
*ε*	Resolution of final tuning	10^–3^
*M*_*c*_	Multiplication factor for enabling sensitivity update using Broyden formula	10

### Verification test cases

**Fig. 11:**
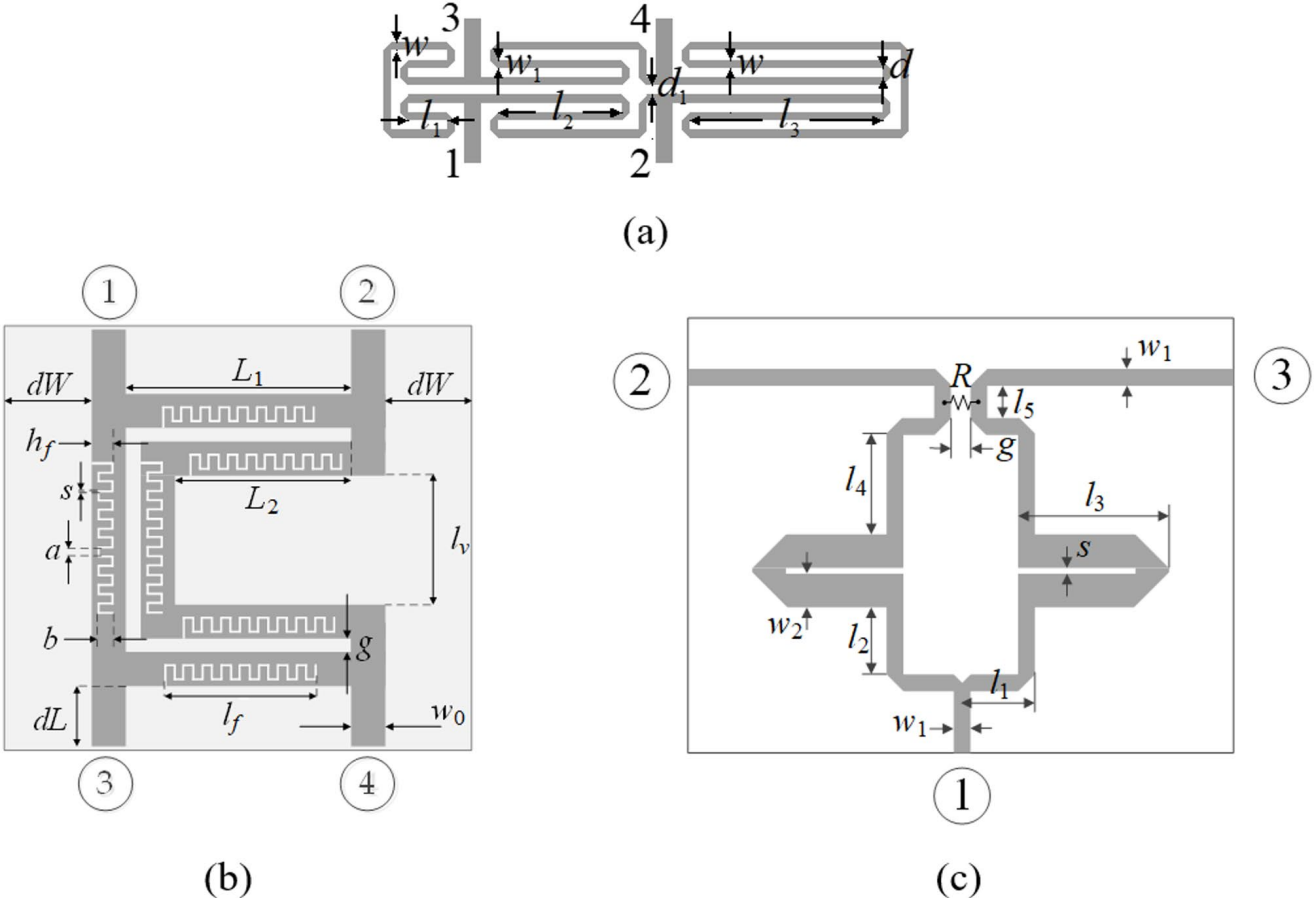
Test circuits: (**a**) a compact rat-race coupler (Circuit I)^55^, (**b**) a rat-race coupler with defected microstrip line (Circuit II)^56^, (**c**) a dual-band power divider (Circuit III)^57^.

**Fig. 12:**
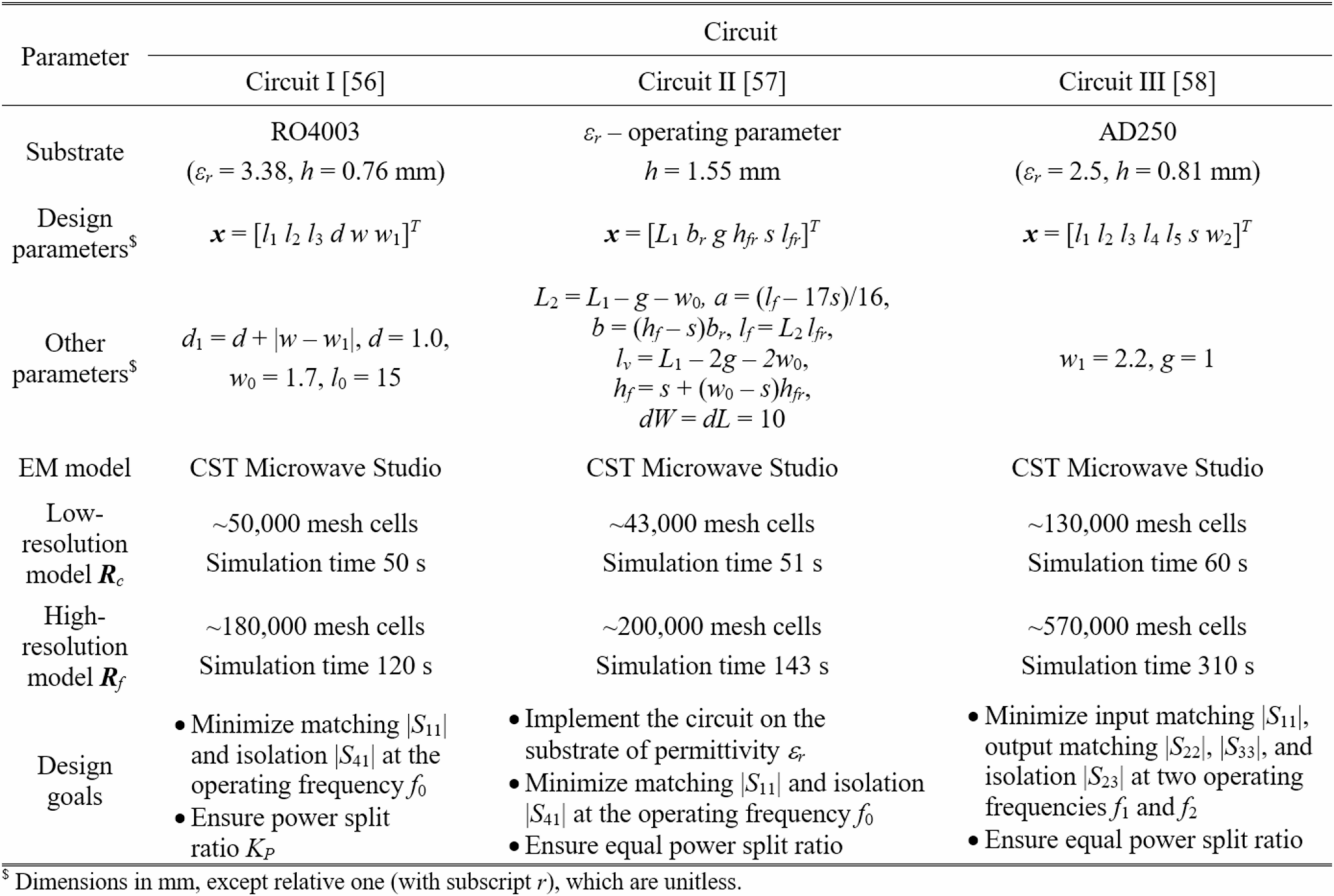
Parameters of circuits of Fig. 11.

**Fig. 13:**
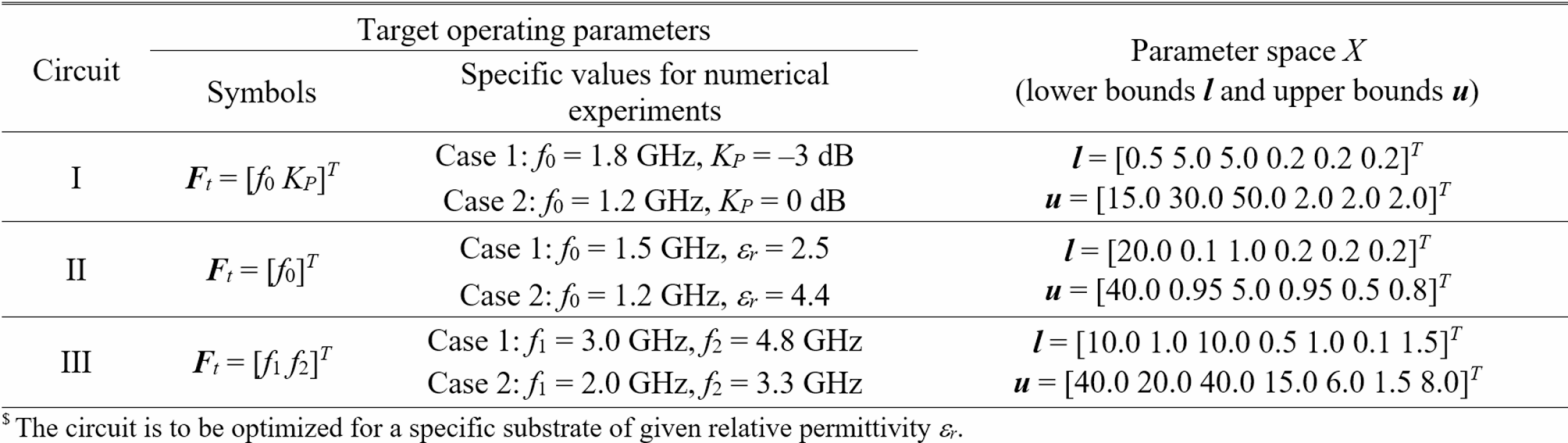
Design objectives and search spaces for circuits of Fig. 11.

The microwave circuits utilized to validate our algorithm are shown in Fig. 11. Their parameters and design objective can be found in Fig. 12, whereas the target operating parameters are enlisted in Fig. 13. It is important to emphasize that the search spaces are vast: the mean ratio between the upper and lower bounds exceeds thirteen for Circuit I, five for Circuit II, and twelve for Circuit III.

### Benchmark procedures

Circuits I through III are optimized using our algorithm and several benchmark procedures outlined in Table 3. To verify the ability to perform global search and sustain repeatability of solutions, the proposed procedures were run ten times.

The particular choice of benchmark methods has been made to corroborate and illustrate certain qualities of the presented methodology. A comparison with nature-inspired algorithms aims at demonstrating computational advantages of incorporating simplex-anchored regression models as well as conducting the search using the operating parameters rather than complete characteristics. Note that the computational budgets of PSO and DE are set at only 500 objective function calls (Version I) and 1,000 (Version II). These numbers are very conservative yet result in considerable CPU times of several days per algorithm run. The employment of the TR routine (which is a local procedure) has been dictated by the need to confirm multimodality of the our test cases. We also use conventional machine learning optimization with kriging interpolation surrogates and infill criterion based on expected objective function improvement. Meanwhile, the inclusion of the simplex-based algorithm using only high-resolution EM simulations enables verification of the speedup obtained by the variable-resolution approach, as well as determination of whether conducting global search using low-fidelity simulations may have any adverse effects on reliability.

**Table 3:** Algorithm setup (proposed and benchmark).

Algorithm	Name	Outline
This study	Variable-resolution simplex-based global search with gradient-based fine tuning (Sect. 2)	Control parameters: *F*_max_ = 0.2 GHz, *α* = 0.2, *γ* = 0.5, *D*_min_ = 1, *ε* = 10^–3^ and *M*_*c*_ = 10 (cf. Table 2)
I	Particle swarm optimizer (PSO)	Swarm size *N* = 10, number of iterations: 50 (Version I) and 100 (Version II); conventional parameter setup (*χ* = 0.73, *c*_1_ = *c*_2_ = 2.05) [54];
II	Differential evolution (DE)	Population size *N* = 10, number of iterations set to 50 (Version I) and 100 (Version II); conventional parameter setup (*CR* = 0.5, *F* = 1) [55];
III	Trust-region (TR) gradient-based optimizer	Random initialization, Jacobian matrix estimated by finite differentiation, termination: convergence in argument
IV	Machine learning optimization	Initial surrogate set up to ensure relative RMS error not higher than 20% with the maximum number of training samples equal to 400; Infill criterion: minimization of the predicted objective function; surrogate model constructed at the level of circuit’s frequency characteristics.
V	Simplex-based global search with gradient-based fine tuning	Algorithm setup: the same as for the proposed approach, except that the search process is conducted using ***R***_*f*_

### Numerical results. comparative analysis

**Fig. 14:**
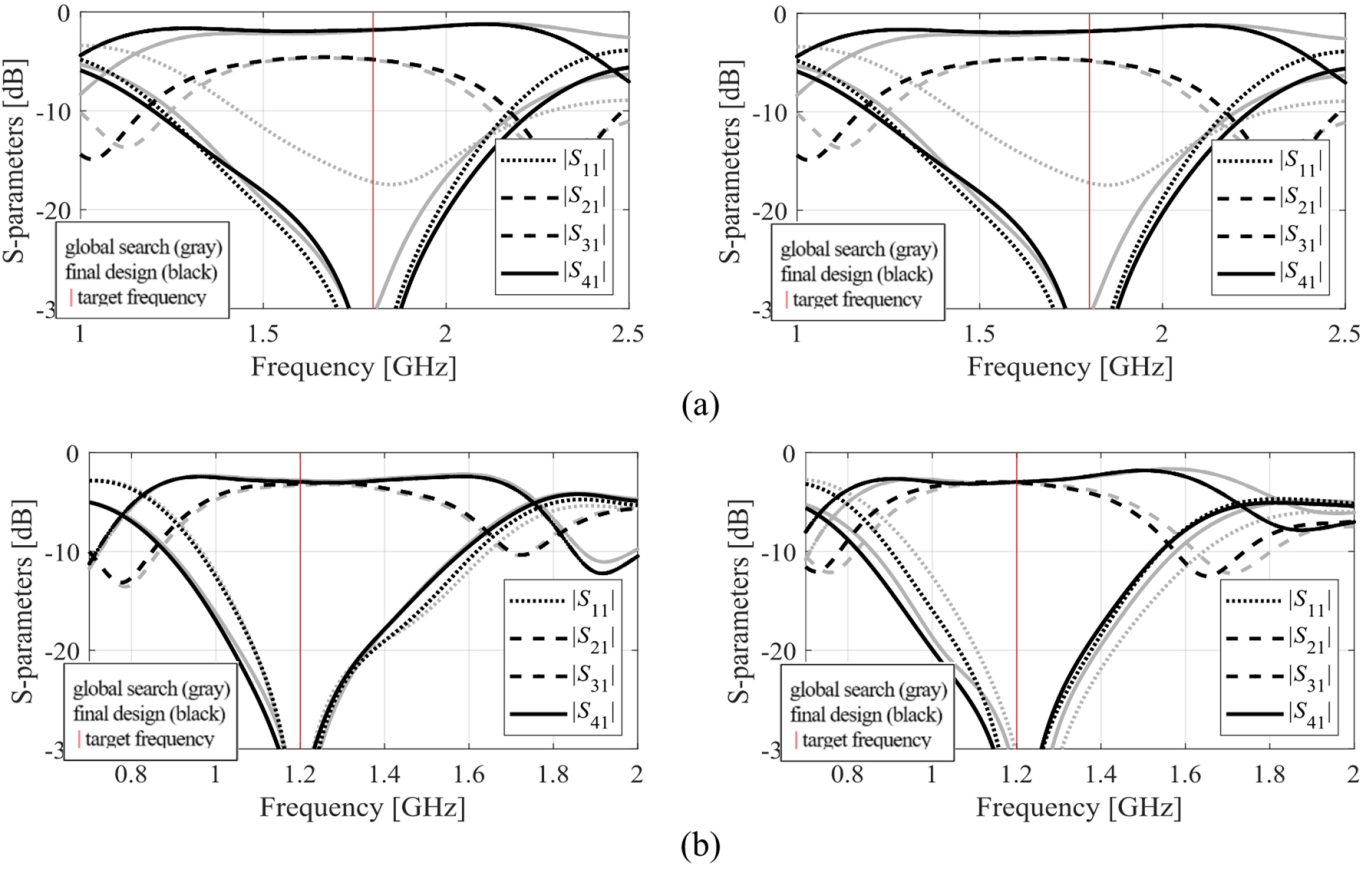
Circuit I: *S*-parameters at the designs found using our algorithm for selected runs: (**a**) Case 1, (**b**) Case 2.

**Fig. 15:**
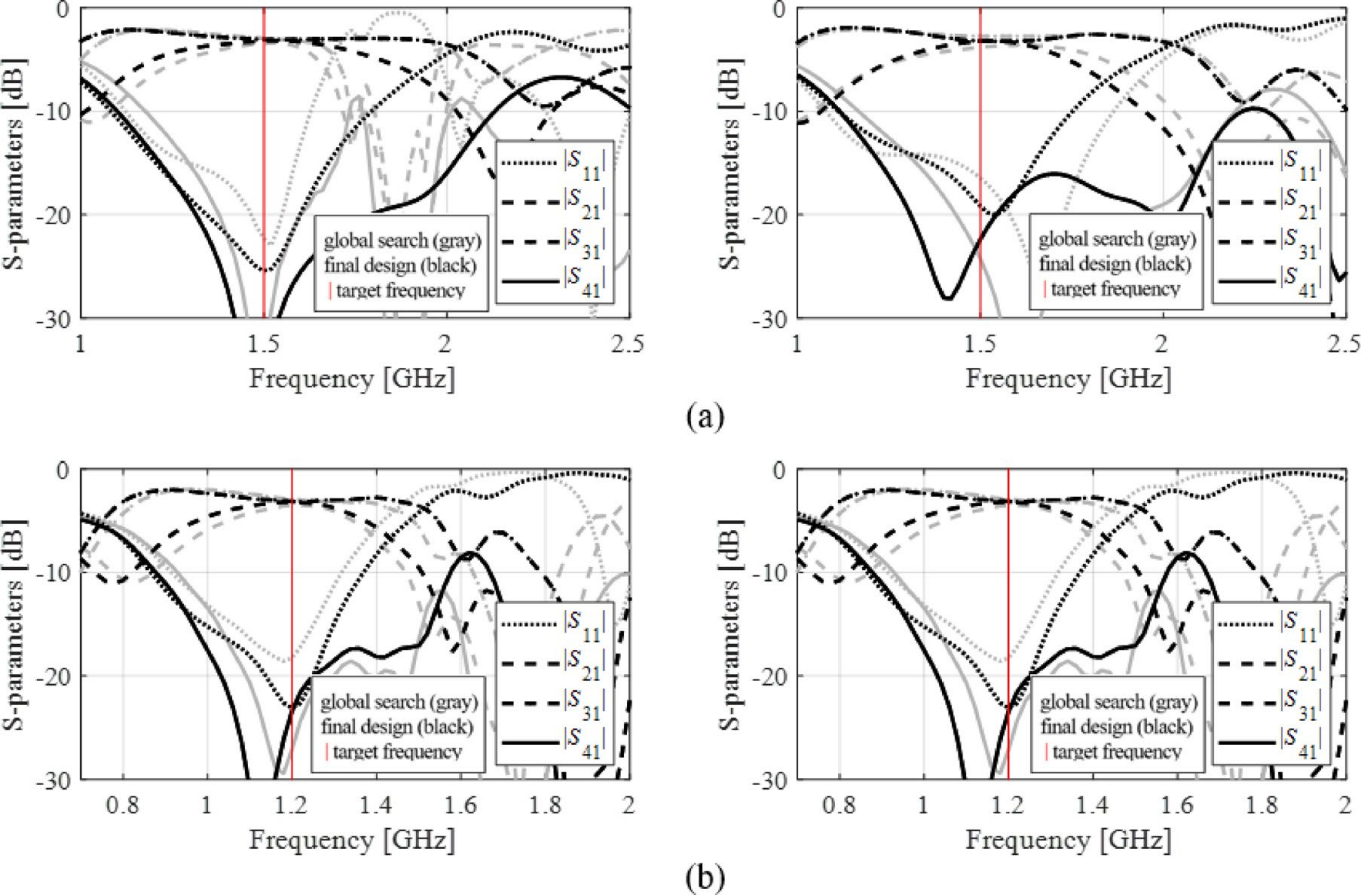
Circuit II: *S*-parameters at the designs found using our algorithm for selected runs: (**a**) Case 1, (**b**) Case 2.

**Fig. 16:**
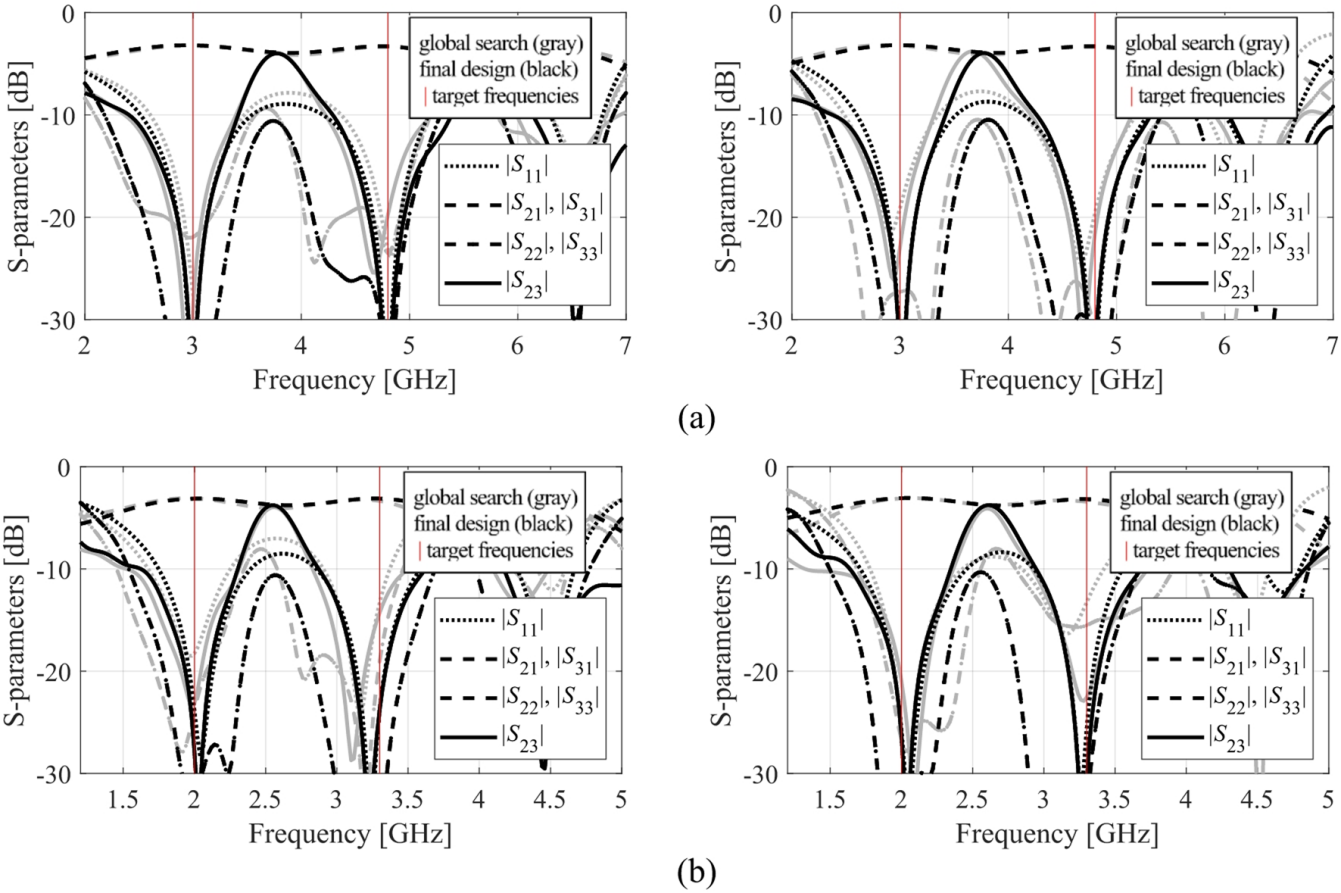
Circuit III: *S*-parameters at the designs found using our algorithm for selected runs: (**a**) Case 1, (**b**) Case 2.

The results produced for Circuits I through III are encapsulated in (Tables 4, 5, 6). Shown is the value of the objective function, the algorithm running cost, and the success rate, i.e., a fraction of runs for which ||***f***(x^*^) – ***f***_*t*_)|| ≤ *F*_max_. The results are averaged over ten runs carried out for each procedure. The circuit responses (*S*-parameters versus frequency) at the conclusion of the global search and at the final design are shown in Figs. 14, 15, 16 for all three circuits.

The remaining part of this section discusses the results focusing on reliability, design quality, and cost efficiency. The speedup obtained by incorporating variable-fidelity models and whether it might be detrimental to reliability are the two particularly important points of this analysis.

As mentioned earlier, in this work, the reliability is assessed using the success rate, measured as the fraction of algorithm runs for which ||***f***(x^*^) – ***f***_*t*_) ≤ *F*_max_ (out of ten runs executed). The proposed technique exhibits perfect rate of 10/10 for all considered test cases. Due to a large extent of the numerical experiments (three circuits, two specifications for each), this result confirms the global search capability. The same score was obtained by the last benchmark method (simplex-based search at high-resolution level); however, at considerably higher computational expenses. This aspect will be discussed in more detail later on. On the other hand, the success rate of randomly-initialized gradient search is low, only 5/10 on the average, which is indicative of multimodality of the problems included in the testbed. Finally, the performance of the bio-inspired algorithms (PSO and DE) is better, yet not perfect. The average success rate thereof is slightly around 9/10 for the first version (500 objective function calls), and about 9.5/10 for the second version (1,000 function calls). The machine learning algorithm yields significantly better results, comparable with the proposed technique. While ensuring the perfect success rate of 10/10, its computational efficiency is; however, considerably worse (the typical costs between 400 and 500 EM simulations). This demonstrates the ability of the nature-inspired approach to identify global optima but also insufficiency of the given budget. Based on the success rate and the objective function values, the minimum budget necessary for this method to yield consistent results is at least 2,000 objective function evaluations. Clearly, from practical point of view, anything beyond 1,000 is hardly acceptable: for the considered circuits, it translates into the running time of two to four days of the computing time.

**Table 4:** Results for circuit I.

	Algorithm	This work	PSO		DE		TR gradient-based algorithm	Machine learning	Simplex-based procedure using high-resolution EM model
			50 iterations	100 iterations	50 iterations	100 iterations			
Case 1 *f*_0_ = 1.8 GHz, *K*_*P*_ = − 3 dB	Objective function [dB]	–32.9	–24.8	–34.0	–22.8	–33.2	–18.7	–31.8	–33.6
	Computational cost^$^	60.2	500	1,000	500	1,000	102.8	473.2	74.6
	Success rate^#^	10/10	9/10	10/10	8/10	10/10	6/10	10/10	10/10
Case 2 *f*_0_ = 1.2 GHz, *K*_*P*_ = 0 dB	Objective function [dB]	–37.5	–23.7	–36.2	–25.8	–35.6	48.3	–34.1	–38.3
	Computational cost^$^	43.1	500	1,000	500	1,000	68.7	435.7	55.7
	Success rate^#^	10/10	9/10	10/10	9/10	10/10	5/10	10/10	10/10

^$^The number of high-resolution EM analyses.

^#^A fraction of runs such that ||***f***(x^*^) – ***f***_*t*_) ≤ *F*_max_.

**Table 5:** Results for circuit II.

	Algorithm	This work	PSO		DE		TR gradient-based algorithm	Machine learning	Simplex-based procedure using high-resolution EM model
			50 iterations	100 iterations	50 iterations	100 iterations			
Case 1 *f*_0_ = 1.5 GHz, *ε*_*r*_ = 2.5	Objective function [dB]	–18.2	–17.6	–19.2	–17.9	–18.3	1.8	–17.9	–18.6
	Computational cost^$^	63.1	500	1,000	500	1,000	77.0	482.3	76.9
	Success rate^#^	10/10	10/10	10/10	9/10	10/10	5/10	10/10	10/10
Case 2 *f*_0_ = 1.2 GHz, *ε*_*r*_ = 4.4	Objective function [dB]	–18.5	–19.4	–22.5	–18.9	–21.5	7.6	–18.5	–19.4
	Computational cost^$^	55.8	500	1,000	500	1,000	83.8	456.3	69.8
	Success rate^#^	10/10	9/10	10/10	9/10	10/10	5/10	10/10	10/10

^$^The number of high-resolution EM analyses.

^#^A fraction of runs such that ||***f***(x^*^) – ***f***_*t*_) ≤ *F*_max_.

**Table 6:** Results for circuit III.

	Algorithm	This work	PSO		DE		TR gradient-based algorithm	Machine learning	Simplex-based procedure using high-resolution EM model
			50 iterations	100 iterations	50 iterations	100 iterations			
Case 1 *f*_1_ = 3.0 GHz, *f*_2_ = 4.8 GHz	Objective function [dB]	–34.9	–19.6	–19.8	–19.9	–21.5	–12.3	–35.6	–33.8
	Computational cost^$^	60.6	500	1,000	500	1,000	95.1	433.6	82.0
	Success rate^#^	10/10	8/10	9/10	9/10	9/10	2/10	10/10	10/10
Case 2 *f*_1_ = 2.0 GHz, *f*_2_ = 3.3 GHz	Objective function [dB]	–25.8	–18.8	–19.7	–19.1	–20.0	–20.6	–28.3	–25.9
	Computational cost^$^	62.7	500	1,000	500	1,000	93.8	458.2	84.6
	Success rate^#^	10/10	8/10	9/10	8/10	9/10	7/10	10/10	10/10

^$^The number of high-resolution EM analyses.

^#^A fraction of runs such that ||***f***(x^*^) – ***f***_*t*_) ≤ *F*_max_.

The design quality, assessed by means of the objective function is comparable to what has been produced by the benchmark methods (more specifically, the successful runs thereof). This means that expediting the optimization process by the algorithmic means implemented here is not detrimental to quality (note that the differences at the level of a few decibels can be considered insignificant for the objective function values below − 25 dB or so). It should also be stressed that the essential benefit of the presented methodology is improved robustness as pointed out in the previous paragraph.

Cost efficiency of our technique can be accessed as excellent. The average cost CPU expenses correspond to only 58 high-resolution EM analyses, less than the cost of gradient-based search (the average of 86 EM analyses). While this may seem surprising as the methodology of Sect. 2 also includes local tuning as the last stage, one needs to remember that the final tuning is initiated from a point already close to the target (owing to the preceding global search stage), and the process is expedited by employing the Broyden update when close to convergence. When compared to global optimization methods, the proposed approach offers 94% savings with respect to PSO and DE, 87% savings over the machine learning algorithm, and 22% savings over the simplex-based algorithm operating at the high-resolution EM level. The latter demonstrates an important role variable-resolution models: the incorporation of low-fidelity simulations for parameter space pre-screening and global optimization stage translates into significant speedup without degrading the design quality. Also, recall that the low-fidelity model does not undergo any correction; its inaccuracy is compensated for at the final tuning stage. This simplifies implementation of the method.

Apart from variable-fidelity modeling, the two major acceleration factors are related to utilization of the simplex-based surrogates in conjunction with processing the operating figures of the circuit rather than its entire frequency characteristics. The latter capitalizes on weakly-nonlinear relationship between operating figures (center frequency, power split ratio, etc.) and geometry parameters. Meanwhile, establishing and updating the simplex vertices (training dataset for the surrogate) is computationally cheap, which reduces the overall optimization costs even further.

The demonstrated performance of the proposed variable-resolution framework is indicative of its suitability for solving global parameter tuning tasks in microwave engineering. Its excellent computational efficiency makes it a viable alternative to bio-inspired and surrogate-assisted methods. A potential restriction is associated with the parameter space size. If the parameter ranges are excessively large so that most designs are of poor quality, identification of good-quality samples to serve as initial simplex vertices (cf. Section 2.3) may be severely impeded. In particular, the computational cost of the pre-sampling stage may increase dramatically. However, in a well-posed design problem, the parameter space is normally established using engineering insight with parameter ranges set up reasonably. Furthermore, conducting the search process using operating parameters implicitly regularizes the merit function, which mitigates the mentioned issue. In particular, for problems considered in this study, the search spaces are vast: the ratio between the upper and lower parameter bound is about ten on average; yet, the proposed technique successfully identified satisfactory design in all cases. Needless to say, carrying out global optimization over large parameter spaces would be detrimental to any search procedure, including nature-inspired algorithms and even more machine-learning routines involving surrogate modeling techniques.

Supplementary experiments were conducted to assess the impact of control parameters, specifically *F*_max_, *α*, *γ*, and *D*_min_ (cf. Table 3). For the sake of brevity, the experiments are carried out for Circuit I (Case I). The results are encapsulated in Table 7. As expected, varying the parameters has a minor effect on the algorithm’s performance. For all cases, the perfect success rate is maintained. There are noticeable variations concerning the achieved objective function value (within the ± 1dB limit), and the computational cost (within ± 5 high-fidelity EM simulations). Reducing *F*_max_ and *D*_min_ is more restrictive for the global search stage, which translates into its slightly higher cost. At the same time, the local tuning cost is typically faster, so that the global cost is comparable. Changing *α* has almost no effect as expected, whereas reducing *γ* (the simplex reduction factor) generally leads to a faster convergence of the global search stage, which may compromise the reliability and increase the cost of the local tuning phase. It can be observed that relaxing convergence criteria for the global search stage makes the local tuning step more demanding (and longer) with the overall cost being comparable.

### Experimental validation

**Fig. 17:**
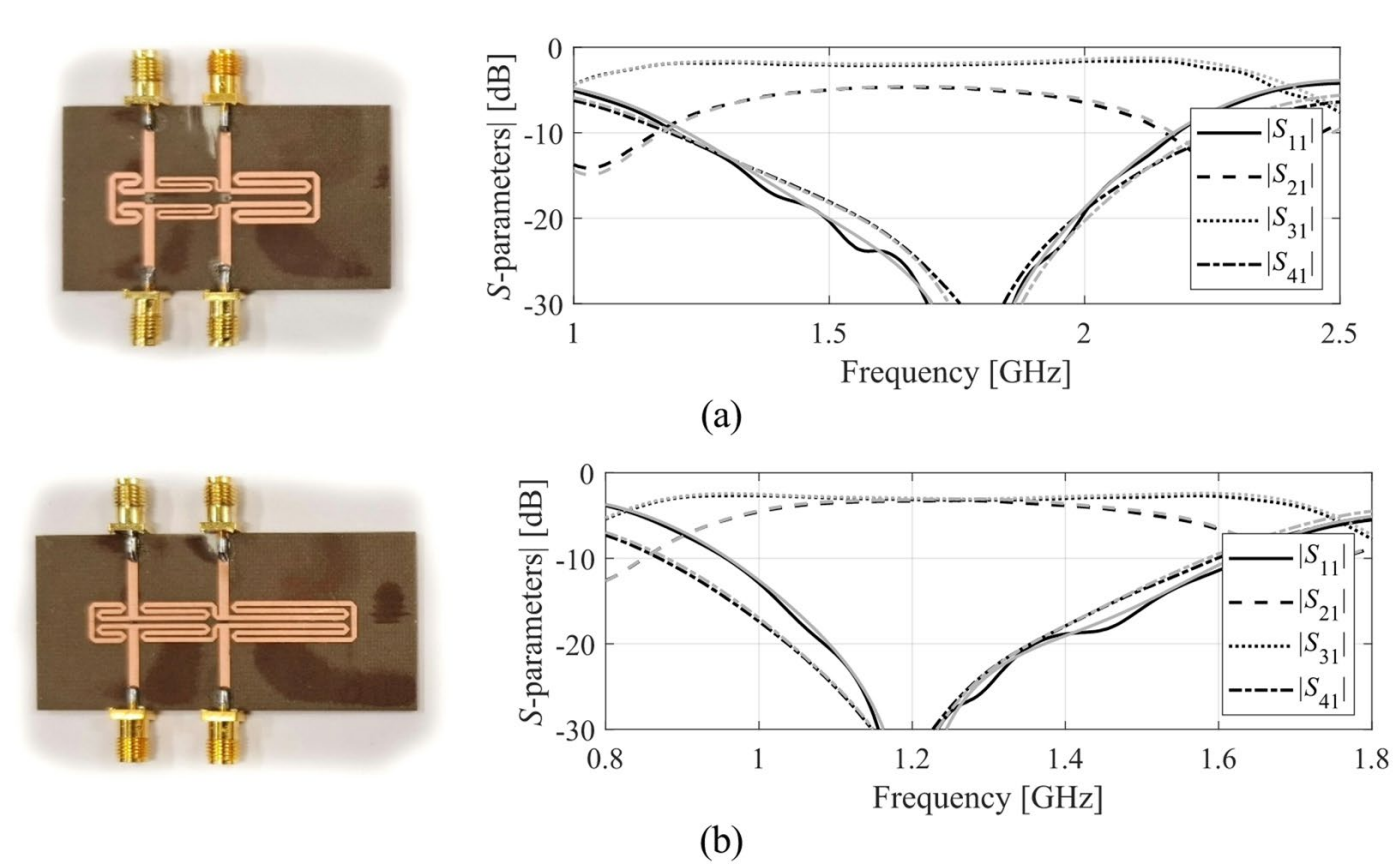
Circuit I prototypes and EM-simulated (gray) versus measured (black) frequency characteristics: (**a**) Case 1, (**b**) Case 2 (both corresponding to the left-hand-side panels of Fig. 14).

**Fig. 18:**
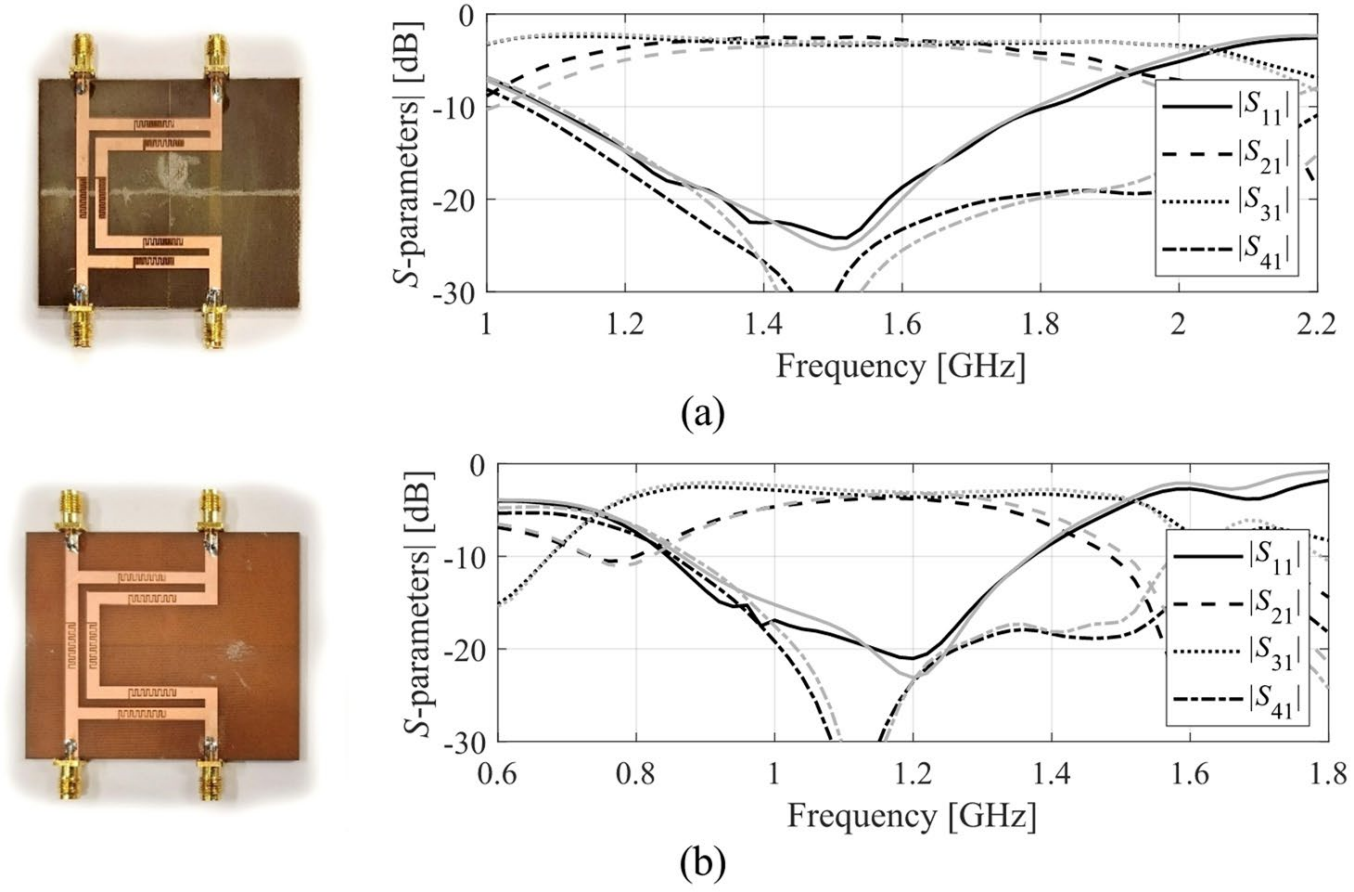
Circuit II prototypes and EM-simulated (gray) versus measured (black) frequency characteristics: (**a**) Case 1, (**b**) Case 2 (both corresponding to the left-hand-side panels of Fig. 15).

**Fig. 19:**
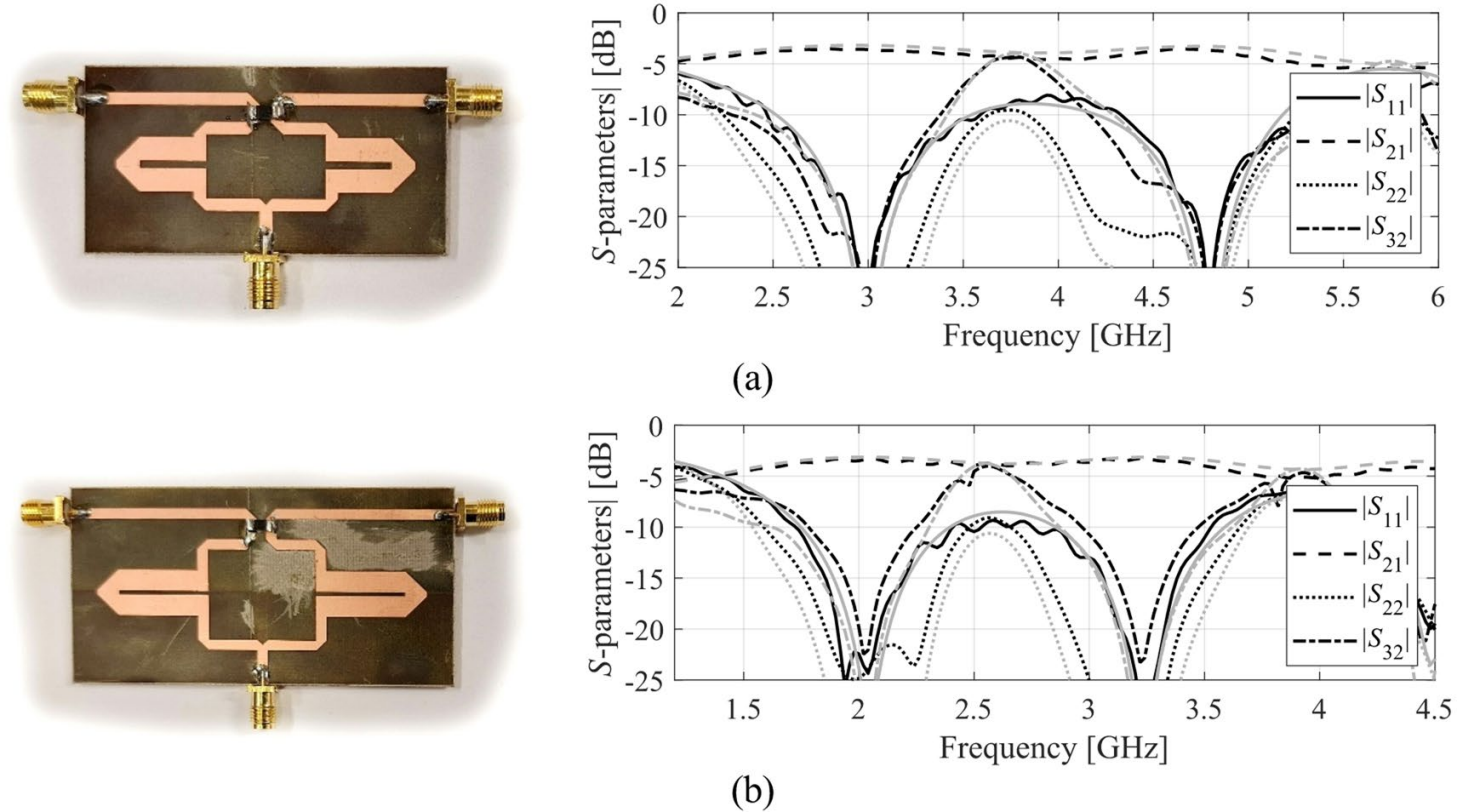
Circuit III prototypes and EM-simulated (gray) versus measured (black) frequency characteristics: (**a**) Case 1, (**b**) Case 2 (both corresponding to the left-hand-side panels of Fig. 16).

Additional verification has been arranged as experimental validation of selected optimum designs found using the proposed procedure. The circuits manufactured and measured correspond to those presented on the left panels of Figs. 14 (Circuit I), 15 (Circuit II), and 16 (Circuit III). The circuit prototypes as well as simulated and measured responses are included in Figs. 17 and 18, and Fig. 19 for Circuits I through III, respectively. The agreement between the datasets is satisfactory. Small deviations are due to manufacturing and assembly inaccuracies and the effects of connectors.

**Table 7:** Impact of control parameter setup (Circuit I, case I).

Control parameter				Results		
*F*_max_	*α*	*γ*	*D*_min_	Objective function [dB]	Computational cost	Success rate
0.2	0.2	0.5	1	–18.2	63.1	10/10
0.2	0.2	0.33	2	–18.8	65.2	10/10
0.2	0.1	0.5	1	–18.1	60.8	10/10
0.2	0.1	0.33	2	–17.9	68.9	10/10
0.1	0.2	0.5	1	–18.2	64.3	10/10
0.1	0.2	0.33	2	–19.0	62.1	10/10
0.1	0.1	0.5	1	–17.5	70.1	10/10
0.1	0.1	0.33	2	–18.6	68.5	10/10

## Conclusion

This research introduced a new approach to the globalized parameter adjustment of microwave circuits. Our method has been developed to ensure dependability and computational efficiency. The keystone of the algorithm is regression models constructed using sparse training data allocated in the form of simplexes. The models represent the circuit’s operating parameters rather than its complete frequency responses, which regularizes the objective function and improves the predictive power of the surrogates. Updating the simplex involves just one EM analysis per iteration, which leads to considerably reduced optimization costs. Meanwhile, a global search is executed using low-resolution EM simulations to yield further savings. The final outcome is determined based on local tuning expedited by the Broyden updating formula when close to convergence. To ensure reliability, this last stage is conducted using high-resolution EM analysis.

The presented framework has been validated using several microstrip circuits. For each circuit, two design scenarios were considered, different concerning the target operating parameters. Despite large parameter spaces, our technique demonstrated excellent performance. It exhibits a perfect success rate, that is, a satisfactory design is identified in each run. Furthermore, the computational efficiency is remarkable with the optimization process cost corresponding to only about sixty EM simulations, which is less than for local gradient-based tuning. The savings over nature-inspired optimization (here, PSO and DE) are as high as 94%, whereas the speedup over the simplex-based routine operating at high-resolution EM level is 22%. The aforementioned acceleration is achieved without compromising the design quality.

A potential limitation of our framework is that the search process (in particular, its global optimization stage) is conducted by handling the operating parameters. For excessively large parameter spaces, identification of a required number of designs with extractable operating parameters may become an issue, which would manifest itself through an increased cost of the pre-sampling stage. On the other hand, poor-quality designs are rejected during the pre-screening step, which facilitates determination of more promising regions. Furthermore, a reasonable setup of the parameter bounds using engineering experience makes the optimization process more failure-proof and reduces the likelihood of incurring excessive cost. Further work will be devoted to investigate these aspects more closely. Notwithstanding, the presented results suggest that the proposed procedure has a potential to replace or complement conventional optimization procedures, e.g., bio-inspired metaheuristics, in microwave engineering practice, especially whenever computational efficiency is of high priority. Also, we will extend the applicability range of the framework to more complex structures, including multi-layer devices.

## Data Availability

The datasets used and/or analyzed during the current study are available from the corresponding author on reasonable request.

## References

[1] Hagag, M. F., Zhang, R. & Peroulis, D. „High-performance tunable narrowband SIW cavity-based quadrature hybrid coupler. *IEEE Microw. Wirel. Comp. Lett.* **29** (1), 41–43 (2019).

[2] Li, Q., Chen, X., Chi, P. & Yang, T. „Tunable Bandstop filter using distributed coupling microstrip resonators with capacitive terminal. *IEEE Microw. Wirel. Comp. Lett.* **30** (1), 35–38 (2020).

[3] Sheikhi, A., Alipour, A. & Mir, A. „Design and fabrication of an ultra-wide stopband compact bandpass filter. *IEEE Trans. Circuits Syst. II: Express Briefs*. **67** (2), 265–269 (2020).

[4] Firmansyah, T., Alaydrus, M., Wahyu, Y., Rahardjo, E. T. & Wibisono, G. A highly independent multiband bandpass filter using a multi-coupled line stub-SIR with folding structure. *IEEE Access.* **8**, 83009–83026 (2020).

[5] Chen, S. et al. A frequency synthesizer based microwave permittivity sensor using CMRC structure. *IEEE Access.* **6**, 8556–8563 (2018).

[6] Deng, J., Li, M., Sun, D., Guo, L. & Ma, X. „Compact dual-band inverted-microstrip ridge gap waveguide bandpass filter. *IEEE Trans. Microw. Theory Techn*. **68** (7), 2625–2632 (2020).

[7] Koziel, S. & Abdullah, M. Machine-learning-powered EM-based framework for efficient and reliable design of low scattering metasurfaces. *IEEE Trans. Microw. Theory Techn*. **69** (4), 2028–2041 (2021).

[8] Li, Y., Ren, P. & Xiang, Z. „A dual-passband frequency selective surface for 5G communication. *IEEE Antennas Wirel. Propag. Lett.* **18** (12), 2597–2601 (2019).

[9] Li, H., Jiang, Y., Ding, Y., Tan, J. & Zhou, J. Low-sidelobe pattern synthesis for sparse conformal arrays based on PSO-SOCP optimization. *IEEE Access.* **6**, 77429–77439 (2018).

[10] Pietrenko-Dabrowska, A. & Koziel, S. „Fast design closure of compact microwave components by means of feature-based metamodels. *Electronics* **10**, (2021).

[11] Li, X. & Luk, K. M. The grey Wolf optimizer and its applications in electromagnetics. *IEEE Trans. Ant Prop.* **68** (3), 2186–2197 (2020).

[12] Luo, X., Yang, B. & Qian, H. J. Adaptive synthesis for resonator-coupled filters based on particle swarm optimization. *IEEE Trans. Microw. Theory Techn*. **67** (2), 712–725 (2019).

[13] Ding, D., Zhang, Q., Xia, J., Zhou, A. & Yang, L. Wiggly parallel-coupled line design by using multiobjective evolutionay algorithm. *IEEE Microw. Wirel. Comp. Lett.* **28** (8), 648–650 (2018).

[14] Greda, L. A., Winterstein, A., Lemes, D. L. & Heckler, M. V. T. Beamsteering and beamshaping using a linear antenna array based on particle swarm optimization. *IEEE Access.* **7**, 141562–141573 (2019).

[15] Cui, C., Jiao, Y. & Zhang, L. „Synthesis of some low sidelobe linear arrays using hybrid differential evolution algorithm integrated with convex programming. *IEEE Ant Wirel. Propag. Lett.* **16**, 2444–2448 (2017).

[16] Zheng, T. et al. IWORMLF: improved invasive weed optimization with random mutation and Lévy flight for beam pattern optimizations of linear and circular antenna arrays. *IEEE Access.* **8**, 19460–19478 (2020).

[17] Liang, S. et al. „Sidelobe reductions of antenna arrays via an improved chicken swarm optimization approach. *IEEE Access.* **8**, 37664–37683 (2020).

[18] Li, W., Zhang, Y. & Shi, X. „Advanced fruit fly optimization algorithm and its application to irregular subarray phased array antenna synthesis. *IEEE Access.* **7**, 165583–165596 (2019).

[19] Goudos, S. K., Yioultsis, T. V., Boursianis, A. D., Psannis, K. E. & Siakavara, K. Application of new hybrid Jaya grey Wolf optimizer to antenna design for 5G communications systems. *IEEE Access.* **7**, 71061–71071 (2019).

[20] Liu, F., Liu, Y., Han, F., Ban, Y. & Jay Guo, Y. Synthesis of large unequally spaced planar arrays utilizing differential evolution with new encoding mechanism and cauchy mutation. *IEEE Trans. Antennas Propag.* **68** (6), 4406–4416 (2020).

[21] Kovaleva, M., Bulger, D. & Esselle, K. P. „Comparative study of optimization algorithms on the design of broadband antennas. *IEEE J. Multiscale Multiphysics Comp. Techn*. **5**, 89–98 (2020).

[22] Bai, Y., Xiao, S., Liu, C. & Wang, B. A hybrid IWO/PSO algorithm for pattern synthesis of conformal phased arrays. *IEEE Trans. Antennas Propag.* **61** (4), 2328–2332 (2013).

[23] Li, Y., Xiao, S., Rotaru, M. & Sykulski, J. K. A dual kriging approach with improved points selection algorithm for memory efficient surrogate optimization in electromagnetics. *IEEE Trans. Magn.* **52** (3), 1–4, (2016).

[24] Ogut, M., Bosch-Lluis, X. & Reising, S. C. „A deep learning approach for microwave and millimeter-wave radiometer calibration. *IEEE Trans. Geoscience Remote Sens.* **57** (8), 5344–5355 (2019).

[25] Jacobs, J. P. Characterization by Gaussian processes of finite substrate size effects on gain patterns of microstrip antennas. *IET Microwaves Ant Prop.* **10** (11), 1189–1195 (2016).

[26] Zhang, Z., Cheng, Q. S., Chen, H. & Jiang, F. An efficient hybrid sampling method for neural network-based microwave component modeling and optimization. *IEEE Microw. Wirel. Comp. Lett.* **30** (7), 625–628 (2020).

[27] Yu, X. et al. „A method to select optimal deep neural network model for power amplifiers. *IEEE Microw. Wirel. Comp. Lett.* **31** (2), 145–148 (2021).

[28] Couckuyt, I., Declercq, F., Dhaene, T., Rogier, H. & Knockaert, L. Surrogate-based infill optimization applied to electromagnetic problems. *Int. J. RF Microw. Computt -Aided Eng.* **20** (5), 492–501 (2010).

[29] Torun, H. M. & Swaminathan, M. High-dimensional global optimization method for high-frequency electronic design. *IEEE Trans. Microw. Theory Techn*. **67** (6), 2128–2142 (2019).

[30] Liu, B., Koziel, S. & Zhang, Q. A multi-fidelity surrogate-model-assisted evolutionary algorithm for computationally expensive optimization problems. *J. Comp. Sc*. **12**, 28–37 (2016).

[31] Taran, N., Ionel, D. M. & Dorrell, D. G. Two-level surrogate-assisted differential evolution multi-objective optimization of electric machines using 3-D FEA, *IEEE Trans. Magn.* **54** (11), (2018).

[32] Koziel, S. & Pietrenko-Dabrowska, A. *Performance-driven Surrogate Modeling of high-frequency Structures* (Springer, 2020).

[33] Pietrenko-Dabrowska, A. & Koziel, S. Antenna modeling using variable-fidelity EM simulations and constrained co-kriging. *IEEE Access.* **8** (1), 91048–91056 (2020).

[34] Pietrenko-Dabrowska, A. & Koziel, S. *Response Feature Technology for high-frequency electronics. Optimization, modeling, and Design Automation* (Springer, 2023).

[35] Koziel, S. & Pietrenko-Dabrowska, A. Expedited feature-based quasi-global optimization of multi-band antennas with Jacobian variability tracking. *IEEE Access.* **8**, 83907–83915 (2020).

[36] Koziel, S. & Pietrenko-Dabrowska, A. Fast machine-learning-enabled size reduction of microwave components using response features, *Sc. Rep.* **14**, (2024). 10.1038/s41598-024-73323-w PMC11439033 39341966

[37] Ullah, U., Koziel, S. & Mabrouk, I. B. Rapid re-design and bandwidth/size trade-offs for compact wideband circular polarization antennas using inverse surrogates and fast EM-based parameter tuning. *IEEE Trans. Ant Prop.* **68** (1), 81–89 (2019).

[38] Marler, R. T. & Arora, J. S. The weighted sum method for multi-objective optimization: new insights. *Struct. Multidisc Opt.* **41**, 853–862 (2010).

[39] Mirjalili, S. & Dong, J. S. *Multi-Objective Optimization Using Artificial Intelligence Techniques* (Springer Briefs in Applied Sciences and Technology, 2019).

[40] Koziel, S. & Pietrenko-Dabrowska, A. Recent advances in accelerated multi-objective design of high-frequency structures using knowledge-based constrained modeling approach, *Knowledge Based Syst.* **214** (2021).

[41] Cervantes-González, J. C. et al. Space mapping optimization of handset antennas considering EM effects of mobile phone components and human body. *Int. J. RF Microw. CAE*. **26** (2), 121–128 (2016).

[42] Koziel, S. & Ogurtsov, S. *Antenna Design by simulation-driven optimization. Surrogate-based Approach* (Springer, 2014).

[43] Koziel, S. & Bandler, J. W. A space-mapping approach to microwave device modeling exploiting fuzzy systems, *IEEE Trans. Microwave Theory Tech.* **55** (12), 2539–2547 (2007).

[44] Koziel, S. & Ogurtsov, S. Model management for cost-efficient surrogate-based optimization of antennas using variable-fidelity electromagnetic simulations. *IET Microwaves Ant Prop.* **6** (15), 1643–1650 (2012).

[45] Pietrenko-Dabrowska, A. & Koziel, S. Accelerated gradient-based optimization of antenna structures using multi-fidelity simulation models. *IEEE Trans. Ant Propag.* **69** (12), 8778–8789 (2021).

[46] Koziel, S. & Pietrenko-Dabrowska, A. Rapid multi-objective optimization of antennas using nested kriging surrogates and single-fidelity EM simulation models. *Eng. Comp.* **37** (4), 1491–1512 (2019).

[47] Koziel, S. & Pietrenko-Dabrowska, A. Constrained multi-objective optimization of compact microwave circuits by design triangulation and Pareto front interpolation. *Eur. J. Op Res.* (2021).

[48] Lv, Z., Wang, L., Han, Z., Zhao, J. & Wang, W. Surrogate-assisted particle swarm optimization algorithm with Pareto active learning for expensive multi-objective optimization. *IEEE J. Automatica Sinica*. **6** (3), 838–849 (2019).

[49] Pietrenko-Dabrowska, A. & Koziel, S. „Generalized formulation of response features for reliable optimization of antenna structures, *IEEE Trans. Ant. Propag.* (Early View, 2021).

[50] Koziel, S. & Pietrenko, A. Rapid design centering of multi-band antennas using knowledge-based inverse models and response features, *Knowledge Based Syst.* **252**, (2022).

[51] Conn, A. R., Gould, N. I. M. & Toint, P. L. *Trust Region Methods* (MPS-SIAM Series on Optimization, 2000).

[52] Koziel, S. & Pietrenko-Dabrowska, A. Expedited optimization of antenna input characteristics with adaptive Broyden updates. *Eng. Comp.* **37**, 3, (2019).

[53] Koziel, S. & Pietrenko-Dabrowska, A. Variable-fidelity simulation models and sparse gradient updates for cost-efficient optimization of compact antenna input characteristics. *Sensors* **19**, 8, (2019). 10.3390/s19081806 PMC6515375 30991769

[54] Kennedy, J. & Eberhart, R. C. *Swarm Intelligence* (Morgan Kaufmann, 2001).

[55] Koziel, S. & Pietrenko-Dabrowska, A. Reduced-cost surrogate modeling of compact microwave components by two-level kriging interpolation. *Eng. Opt.* **52** (6), 960–972 (2019).

[56] Phani Kumar, K. V. & Karthikeyan, S. S. „A novel design of rat-race coupler using defected microstrip structure and folding technique, *IEEE Applied Electromagnetics Conf. (AEMC)* pp. 1–2, (2013).

[57] Lin, Z. & Chu, Q. X. „A novel approach to the design of dual-band power divider with variable power dividing ratio based on coupled-lines. *Prog Electromagn. Res.* **103**, 271–284 (2010).

